# Efficient communication over complex dynamical networks: The role of matrix non-normality

**DOI:** 10.1126/sciadv.aba2282

**Published:** 2020-05-27

**Authors:** Giacomo Baggio, Virginia Rutten, Guillaume Hennequin, Sandro Zampieri

**Affiliations:** 1 Department of Information Engineering, University of Padova, via Gradenigo, 6/B I-35131 Padova, Italy.; 2 Gatsby Computational Neuroscience Unit, University College London, London W1T 4JG, UK.; 3 Janelia Research Campus, Howard Hughes Medical Institute, Ashburn, VA, USA.; 4 Computational and Biological Learning Lab, Department of Engineering, University of Cambridge, Cambridge CB2 1PZ, UK.

## Abstract

In both natural and engineered systems, communication often occurs dynamically over networks ranging from highly structured grids to largely disordered graphs. To use, or comprehend the use of, networks as efficient communication media requires understanding of how they propagate and transform information in the face of noise. Here, we develop a framework that enables us to examine how network structure, noise, and interference between consecutive packets jointly determine transmission performance in complex networks governed by linear dynamics. Mathematically, normal networks, which can be decomposed into separate low-dimensional information channels, suffer greatly from readout noise. Most details of their wiring have no impact on transmission quality. Non-normal networks, however, can largely cancel the effect of noise by transiently amplifying select input dimensions while ignoring others, resulting in higher net information throughput. Our theory could inform the design of new communication networks, as well as the optimal use of existing ones.

## Teaser

Non-normal networks efficiently spread information in the face of interference between successive transmissions and readout noise.

## INTRODUCTION

Reliable propagation of information through networks with unreliable nodes is a fundamental problem facing many engineered and natural systems. This includes social networks (*1*, *2*), peer-to-peer networks (*3*), gene regulatory networks (*4*, *5*), power grids (*6*), and brain networks (*7*, *8*), to cite only a few. To engineer better communication networks, make better use of existing ones, or understand how natural (e.g., biological) networked systems function, a theory is needed that relates the network’s connectivity and dynamics to its performance in transmitting information.

Previous work at the interface of network science and information theory has been largely restricted to static, feedforward networks, in which packets of activity travel one after the other through layers of memoryless nodes, with no interference. Examples include classic connectionist work where feedforward “neural” networks are optimized so their outputs retain as much information as possible about their inputs (*9*, *10*). These works have influenced how neuroscientists think about sensory pathways, which resemble layered networks of noisy neurons receiving input packets from body senses (*11*). In particular, the neural representations of visual stimuli that are found along the primate ventral stream are notably similar to those that emerge in deep networks trained on object recognition tasks (*12*). More recent work (*13*) has drawn a link between deep learning (*14*) and the information bottleneck method (*15*), a principled approach to compressive communication. Beyond feedforward networks, the effect of recurrent topologies on information transmission was studied in the context of virtual electrical circuits (*16*), but this was restricted to steady states and therefore disregarded any potential encoding of information in activity transients.

In most real-world scenarios, however, information does not propagate statically (or instantaneously) but dynamically within complex recurrent networks composed of non-memoryless nodes. The inherent dynamics of the network can greatly affect communication performance in ways that remain poorly understood. In (*17*), the authors proposed an analytical framework based atop standard notions of time-delayed mutual information and transfer entropy to quantify the routing of small activity fluctuations propagating on top of oscillatory reference dynamics. While their framework allowed them to identify a generic mechanism capable of generating flexible information-routing patterns in the network, it is based on a small-noise approximation and therefore cannot fully capture the impact of noise on network communication. Moreover, the authors did not systematically study the role of network topology. Harush and Barzel (*18*) investigated the interplay between the network topology and its dynamics. They found that patterns of information are governed by universal laws that depend only on a few relevant parameters of the network dynamics. However, the analysis was carried out in a deterministic setting, and the proposed information transfer metric, which quantifies the sensitivity of a dynamical system to local perturbations, lacks an explicit information-theoretic interpretation. The work in (*19*) used Fisher information theory to quantify the short-term memory storage capacity of networks governed by linear dynamics. In investigating this memory problem, which is a form of network communication through time, the authors were led to study the interactions between single-node dynamics, connectivity, and input statistics, similar to the theory that we develop here. However, the network received a one-dimensional input, and temporal correlations were neglected.

Here, we study the role of graph topology on the quality of information transmission in noisy networks with otherwise simple, linear single-node dynamics. We establish a novel framework for quantifying the maximum amount of information about high-dimensional inputs that can be transmitted reliably through such networks. We apply our framework to various network architectures, ranging from simple, structured networks amenable to analytical derivations to more complex, disordered, and real networks that we investigate numerically. Critically, all the networks that we consider here have memory, from which interference arises between the network’s response to multiple packets transmitted in close succession, and constitutes a source of internal, structured noise. We show that when the amount of noise present in the information channel is large, anisotropic networks that embed directed feedforward pathways perform better than isotropic ones. Mathematically, anisotropic networks correspond to “non-normal” networks [i.e., networks whose adjacency matrices are not normal (*20*, *21*)], whereas isotropic networks to “normal” ones. Moreover, we find that these non-normal networks can even entirely overcome the effect of noise in some limit. Our results provide estimates for the amount of information that a network can propagate and insights into how the propagation of information depends on key network properties. In addition, we discuss how information propagation can be optimized by using specific distributions of input packets. We expect our theory to contribute to understanding the behavior of natural networked systems, which are often found to be strongly non-normal (*22*). Further dissection of the mechanisms at work in natural networks (e.g., single-node dynamics, graph structure, adaptive wiring, etc.) may also suggest better engineered solutions to network communication.

## RESULTS

### Modeling framework

#### Communication through networks

**Fig. 1:**
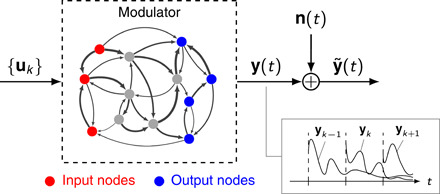
Channel description. A sequence of to-be-transmitted packets of information is encoded in a sequence of random vectors **u***_k_* ∈ ℝ*^m^* independently drawn from an identical encoding probability distribution *p*(**u**). Each **u***_k_* excites the input nodes (red) of a complex dynamical network (Eq. 1). The dynamics of this network act as a “modulator,” producing activation trajectories **y**(*t*) = ∑*_k_**y**_k_*(*t*), with **y***_k_*(*t*) = ***C****e*^***A***(*t*−*kT*)^***B*u***_k_***1**(*t* − *kT*) being the “modulated” waveform corresponding to the input vector *u_k_*, in some output nodes (blue). These are further corrupted by independent Gaussian noise **n**(*t*) before reaching the receiver. The modulator is not memoryless. Because of first-order dynamics in each node of the network, patterns of network activity elicited by previously transmitted packets linger and interfere with the current communication, thus effectively contributing an intrinsic source of structured noise that adds up to the readout noise.

We consider the following model of a communication channel, whereby a sequence of to-be-transmitted packets of information is probabilistically encoded in a sequence of input vectors (Fig. 1). Information transmission occurs via propagation of the inputs through a dynamical network. To obtain analytical, interpretable results that hold for arbitrarily complex graph topologies, we assume minimalistic dynamics for single network nodes: first-order, linear responses to inputs. Specifically, we consider continuous-time, linear dynamical systems of the form

$$\begin{array}{c} \frac{dx(t)}{dt}=Ax(t)+B\;\sum_{k}u_{k}\;\delta (t-\text{kT})\\ y(t)=Cx(t) \end{array}$$ (1)

where **x**(*t*) ∈ ℝ*^n^* denotes the state vector and ***A*** ∈ ℝ^*n*×*n*^ is the state matrix. We restrict our analysis to the case of “stable” network dynamics, whereby responses to transient inputs do not grow unbounded (which would be physically unfeasible) but fade away after some time. Mathematically, this means that we require all eigenvalues of ***A*** to have negative real part.

Each input vector **u***_k_* ∈ ℝ*^m^*, independently drawn from an identical encoding probability distribution *p*(**u**), contains the information carried by the *k*th transmitted packet. Each of these inputs is then delivered as an impulse [here modeled as a Dirac’s delta δ(·)] that excites the network dynamics in Eq. 1. Transmission of successive packets occurs every *T* units of time. The columns of the matrix ***B*** ∈ ℝ^*n*×*m*^ define “input nodes” (red circles in Fig. 1), which are the only ones affected by the impulse. Likewise, a readout matrix ***C*** ∈ ℝ^*p*×*n*^ singles out specific output nodes (blue circles) whose activations **y**(*t*) are transmitted to the receiver, further corrupted by independent Gaussian noise of variance σ^2^. This results in corrupted trajectories $\tilde{y}(t)$, which the receiver could use to reconstruct the corresponding input packets. In our assessment of communication performance, we will consider Shannon’s mutual information as a proxy for reconstruction quality (see below), instead of considering explicit decoding algorithms.

By reducing the complexity of single-node dynamics to simple first-order evolution, Eq. 1 allows us to focus on the effect of network architecture on the quality of information transmission. For example, Eq. 1 is known as a “rate equation” in computational neuroscience, whereby it has been shown to capture key aspects of the dynamics of neuronal networks around fixed points (*23*). Single neurons are often characterized by input/output functions that remain approximately linear over their relevant dynamic range (*24*). In that case, ***A*** represents the matrix of synaptic connection weights, and **x**(*t*) is interpreted as momentary deviations from steady-state firing rates.

Since each network node is governed by first-order dynamics, the network is not memoryless: Activity trajectories elicited by previous communications interfere with (in fact, add linearly to) the network trajectory carrying information about the current input. Thus, for the transmission of a packet at time *t* = 0 (assuming that many packets have already been transmitted), interference contributes an additional source of noise ***i***(*t*), given by

$$i(t)=\sum_{k=1}^{\infty }{Ce}^{A(t+kT)}B\;u_{-k}1(t+\text{kT})$$ (2)

where **1**(·) denotes the unit-step function, defined as **1**(*x*) = 0, if *x* < 0, and **1**(*x*) = 1, otherwise. This phenomenon, known as intersymbol interference in communications, arises in any communication medium that has some form of memory, including networks with node dynamics described by differential equations (*25*).

In the following, we study the combined effects of the network architecture (matrix ***A***), communication time window (*T*), noise level (σ^2^), and encoding of input packets under this communication paradigm. We begin by establishing an analytical framework to characterize the quality of information transmission through the network and highlight the trade-off that arises between sending packets of information at a high temporal rate and the ability for the receiver to accurately reconstruct them. We then summarize our analytical results and illustrate them using appropriate network architectures.

#### Information transmission metrics

To quantify the amount of information that can be propagated through the network channel described above, we use the notion of Shannon’s mutual information between the input packet **u***_k_* and the corresponding noisy network output $\tilde{y}(t)$ observed over the subsequent time interval *kT* ≤ *t* < (*k* + 1)*T*. Denoting by ${\tilde{Y}}_{k}$ this output function (on which intersymbol interference acts as an additional source of noise), and assuming stationarity to drop the *k* subscripts, we can write the mutual information (in bits) between **u** and $\tilde{Y}$ as

$$I_{T}(u,\tilde{Y})=\int p(u)\;du\int p_{T}(\tilde{Y}|u){\text{log}}_{2}\frac{p_{T}(\tilde{Y}|u)}{p_{T}(\tilde{Y})}\;d\tilde{Y}$$ (3)

where the ·*_T_* notation emphasizes the dependence of mutual information on the transmission window (a more formal definition of the integral over functions $\tilde{Y}$ in Eq. 3 is given in note S2). To better use the channel, the sender can use the encoding distribution *p*(**u**) that maximizes the mutual information; this optimum defines an information metric that is independent of the encoding distribution

$$C_{T}=\underset{p(u)}{\text{max}}\;I_{T}(u,\tilde{Y})$$ (4)

With a slight abuse of terminology, we will refer to this metric as information capacity, or simply capacity. Our choice of terminology is motivated by the fact that Eq. 4 coincides with the standard capacity of a digital communication channel, if the channel is memoryless (*26*). We refer to note S2 for further details on the relation between the channel capacity and Eq. 4.

In Eq. 4, the maximization over the encoding distribution *p*(***u***) must be performed with an additional constraint on input power (input covariance). Theoretically, this is required so that the capacity remains finite [the signal-to-noise ratio (SNR) can be made arbitrarily large if inputs can be arbitrarily large, too]. In practice, the nodes of any physical network have limited dynamic range, and therefore, network inputs must be power limited. Here, we consider Gaussian encoding distributions with zero mean and covariance Σ ≽ 0 and input power constraint of the form tr(Σ) ≤ 1 (without loss of generality; cf. note S3).

#### An expression for the information capacity

Our main theoretical result is the following expression for the information capacity (note S2)

$$C_{T}=\frac{1}{2}\underset{\Sigma ≽0,\text{tr}\;\Sigma =1}{\text{max}}{\text{log}}_{2}\frac{\text{det}\;(\sigma^{2}I+OW)}{\text{det}\;(\sigma^{2}I+O(W-B\Sigma B^{⊤}))}$$ (5)

where σ^2^ is the variance of the noise at the receiver, O denotes the observability Gramian over the interval [0, *T*] of the system in Eq. 1, and W is the infinite-horizon controllability Gramian of the dynamics in Eq. 1 discretized with sampling time *T* and input matrix ***B***Σ^1/2^ (*27*). The formal definition of these matrices is reported in Materials and Methods and their properties discussed in note S1. Note that Eq. 5 still involves a (difficult) maximization over the input distribution (via its covariance matrix Σ); in the following, we perform this optimization analytically where possible but otherwise numerically using efficient algorithms (see Materials and Methods).

The information capacity affords a few intuitive properties (cf. note S3). First, $C_{T}$ always grows with increasing SNR = 1/σ^2^. Second, $C_{T}$is a bounded function of *T* that attains its maximum as *T* grows to infinity. This is because, for increasing *T*, (i) network activations left over from previous transmissions have more time to decay away, leading to weaker interference, and (ii) longer stretches of signal are available for decoding, allowing for better estimation of the input signal via additional filtering/denoising. Third, $C_{T}$ cannot decrease if nodes are added to either the set of input nodes or the set of output nodes.

We also note that, in our framework, propagation of information through the network occurs over a finite time window *T*, and packets of information can only be transmitted one at a time. Thus, a more relevant measure of information transmission performance is the number of bits of information about **u** contained in $\tilde{Y}$ per unit time, that is

$$R_{T}=\frac{1}{T}C_{T}$$ (6)

We term this metric information rate. Since the information capacity is bounded (because of output noise and intersymbol interference), ℛ*_T_* always decreases with *T* for large enough *T*. However, we will see that there often exists a nonzero optimal transmission window *T* at which ℛ*_T_* reaches a maximum.

### The limitations of normal networks

**Fig. 2:**
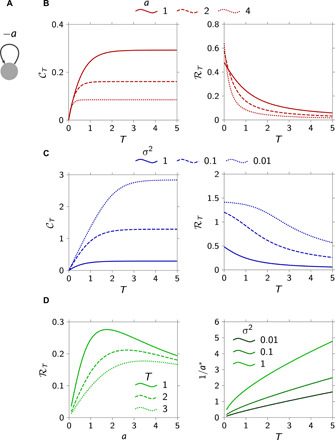
Information transmission through a single node. (**A**) Single-node schematics. (**B** and **C**) Capacity $C_{T}$ and rate ℛ*_T_* as functions of the transmission window *T*, for various values of the decay rate *a* and fixed noise level σ^2^ = 1 (B), and various values of σ^2^ and fixed *a* = 1 (C). (**D**) ℛ*_T_* as a function of the decay time constant 1/*a* exhibits a maximum (1/a*) for any finite value of *T* (left, σ^2^ = 1); this maximum grows with *T* (right).

As we will see later, many high-dimensional networks can be conveniently decomposed as a set of parallel, independent communication channels, each transmitting information about a one-dimensional, scalar quantity. We therefore begin our analysis of the role of connectivity in network communication by an in-depth look at a simple case, that of a single isolated node (Fig. 2A). With ***B*** = ***C*** = Σ = 1, and ***A*** = −*a* < 0 (where 1/*a* > 0 is the node’s decay time constant), Eq. 5 simplifies considerably, yielding the following capacity

$$C_{T}=\frac{1}{2}{\text{log}}_{2}\frac{2a\sigma^{2}+1}{2a\sigma^{2}+e^{-2\mathrm{aT}}}$$ (7)

This expression illuminates some additional properties of the information capacity and its dependence on network parameters. To begin with, $C_{T}$ grows with the allotted transmission window *T* (Fig. 2, B and C, left). Intuitively, this is because increasing the transmission window reduces intersymbol interference, as the node’s activity has more time to decay away before the next packet is transmitted. However, while $C_{T}$ grows linearly with *T* for small increasing *T*, it eventually saturates at a maximum value $\propto {\text{log}}_{2}(1+\frac{1}{2a\sigma^{2}})$ that grows both with the node’s decay time constant (1/*a*) and with the SNR (1/σ^2^). For large enough *T*, the output noise becomes the main factor limiting the capacity and grows increasingly dominant during the transmission of a packet as the node’s activity (the “signal”) decays exponentially over time. Thus, increasing the observation time *T* cannot indefinitely increase the ability of an ideal observer to reconstruct the input packet.

Next, as *T* increases with diminishing returns on the capacity (cf. above), the rate (information per unit time; Eq. 6) is bound to decrease (Fig. 2, B and C, right). Thus, keeping the transmission window very short is the most effective way for a single node to transmit information under time pressure. In this limit, $R_{\text{max}}=\frac{1}{\text{ln}\;2}\frac{a}{1+2a\sigma^{2}}$ bits/s can be transmitted.

In practice, though, transmission windows cannot be made arbitrarily small. For example, visual information conveyed to the brain via the optic nerve fluctuates on a time scale that is limited “at the source” by the rate at which objects move in the scene and by the frequency and speed of saccadic eye movements, which determine an effective sampling frequency (*28*). Thus, we now assume a finite transmission window *T* > 0. In this case, there exists an optimal value of the decay time constant 1/*a* for both ℛ*_T_* (Fig. 2D, left) and $C_{T}$ (not shown). This reflects a trade-off between the noise and intersymbol interference, mathematically evident from Eq. 7, where $C_{T}$ can be seen to go to zero when *a* is either very small or very large. Intuitively, for small decay time constants 1/*a*, intersymbol interference becomes irrelevant, and the information capacity is limited by the effective SNR (1/*a*)/σ^2^, which, in turn, decreases with decreasing 1/*a*. Similarly, for long decay times (increasing 1/*a*), intersymbol interference dominates and ruins the information capacity by letting the summed activities of many previous transmissions pollute the component relevant to the current packet. Thus, the rate (and capacity) is expected to achieve a maximum for some intermediate, optimal value of the decay time constant. Numerically, we find that this optimal time constant scales near-linearly with the transmission window *T* (Fig. 2D, right).

The case of a single-node “network” is, in fact, characteristic of the broader class of so-called normal networks (i.e., networks whose adjacency matrices are normal), which include symmetric, skew-symmetric, and translation-invariant graphs to name only a few examples. In the most favorable communication scenario (i.e., when ***B*** = ***C*** = ***I***), any normal network composed of *n* nodes can be shown to behave like a set of *n* independent scalar information channels (note S5), each corresponding to a specific spatial “mode” of activity at the network level that decays at a specific rate between consecutive transmission events. For example, for a translation-invariant architecture, these channels correspond to Fourier modes of varying spatial frequencies with decay rates that depend on the strength and spatial smoothness of the recurrent interactions (*19*, *29*).

Our mathematical analysis of normal networks shows that, despite their appealing interpretation as sets of parallel communication subchannels, these networks might not be optimally suited for transmitting information. First, as expected from an ensemble of independent scalar subchannels whose rates ℛ*_T_* each decrease with *T* (recall Fig. 2, B and C, right; further examples are given below), multidimensional normal networks with ***B*** = ***C*** = ***I***, too, are best exploited in the limit of very small transmission windows (*T* → 0). As discussed previously, this limit is irrelevant in most applications (where *T* is finite), implying that normal networks would always be suboptimally exploited in practice. Second, we could show that the maximum achievable performance of a normal network does not depend on the fine details of its architecture (e.g., the detailed couplings between nodes) but only on the average decay rate of its nodes (the trace of ***A***). For any choice of ***B*** and ***C***, the information rate of a normal network can never exceed (note S5)

$$R_{\text{max}}=\frac{1}{\text{ln}2}\frac{\text{tr}(A)}{2\sigma^{2}\text{tr}(A)-1}$$ (8)

In particular, the above limit is attained with equality when all nodes are transmitting and receiving packets of information, that is, when ***B*** = ***C*** = ***I***. Critically, there are infinitely many network architectures that share the same tr(***A***) but have otherwise very different geometries. Thus, it would be somewhat unexpected if, among the very large set of all (i.e., normal and non-normal) networks with the same trace, the restricted subset of normal networks achieved the best performance. What is more, Eq. 8 also implies that the maximum rate of any normal network in the low-SNR regime is simply $R_{\text{max}}\approx \frac{1}{2\text{ln}\;2\sigma^{2}}$, which no longer depends on the connectivity matrix ***A***. In other words, no amount of clever structuring of a normal architecture can ever rescue the drop in information rate incurred by a decrease in SNR. These considerations prompted us to study information transmission through more general, non-normal networks.

### Role of non-normality in information transfer

A non-normal network is any network whose connectivity matrix ***A*** is not normal (i.e., ***A*** satisfies ***AA***^†^ ≠ ***A***^†^***A***, where ·^†^ denotes conjugate transposition) (*20*, *21*). Thus, given the equivalence of normal networks with independent parallel channels discussed above, a non-normal network is one that cannot be so decomposed. This implies the existence of effective feedforward pathways, embedded either explicitly at the level of network nodes [i.e., an “anisotropic” tree-like structure that one would notice by looking at the connection graph; (*30*)] or implicitly at the level of orthogonal activity modes that simultaneously involve many nodes [“hidden” feedforward pathways; (*31*–*33*)]. Mathematically, explicit and implicit tree-like structures can both be identified via the Schur decomposition ***A*** = ***U***Δ***U***^†^. If ***A*** is normal, then this decomposition returns a diagonal matrix Δ, with the Schur modes (columns of ***U***) interpreted as separate information channels with decay rates given by the diagonal of Δ. For a non-normal matrix ***A***, the Schur decomposition returns a triangular Δ, the off-diagonal elements of which reveal hidden feedforward connections between the Schur modes.

**Fig. 3:**
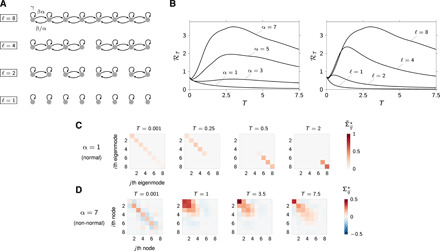
Information rate for chain network. (**A**) Chain network schematics, with parameter values γ = −2.5, β = 1, σ^2^ = 1, ***B*** = ***I***, and ***C*** = ***I***. (**B**) Plot of ℛ*_T_* as a function of *T* for four different values of α (for ℓ = 8) and ℓ (for α = 7). (**C**) Matrix plot of ${\tilde{\Sigma }}^{⋆}$, the optimal input covariance expressed in the eigenbasis of ***A***, for various values of *T*, ℓ = 8, and α = 1 (normal case). The eigenmodes of ***A*** are ordered by increasing decay time constants. (**D**) Matrix plot of the optimal input covariance Σ^⋆^ for various values of *T*, ℓ = 8, and α = 7 (strongly non-normal case).

While it is straightforward to classify a matrix as normal or non-normal, the extent or “degree” to which a matrix departs from normality and how such departure affects the dynamics of the network and communication performance are more difficult to assess. Although several non-normality metrics of either “dynamical” or “algebraic” nature have been proposed in the literature (note S6), there does not exist a unique scalar parameter quantifying the amount of non-normality of general matrices. To address this, we begin with a class of linear graphs whose departure from normality is parameterized by two characteristics that we can choose independently and arbitrarily: the length of the chains embedded in the graph and the directionality of these chains (Fig. 3A). More generally, structural indicators of network non-normality comprise (i) absence of cycles, (ii) low reciprocity of directed edges, and (iii) presence of hierarchical organization (*22*). However, when the network is stable, the strength and length of directional paths in the networks can be regarded as effective indicators of non-normality [cf. note S6 and (*30*)]. The connectivity matrix of the abovementioned networks reads

$$A=[\begin{array}{ccccc} \gamma & \beta /\alpha & 0 & \cdots & 0\\ \alpha \beta & \gamma & \beta /\alpha & \ddots & \vdots \\ 0 & \alpha \beta & \ddots & \ddots & 0\\ \vdots & \ddots & \ddots & \ddots & \beta /\alpha \\ 0 & \cdots & 0 & \alpha \beta & \gamma \end{array}]\in R^{n\times n}$$ (9)

where α, β > 0, and γ < − 2β to enforce stability. The simplicity of this architecture allows us to conveniently decouple the effects of (i) the eigenvalues of ***A*** and (ii) its departure from normality, on the network dynamics (see below). We show later that the insights obtained from this simple structured example topology, especially concerning the role of network non-normality, carry over to higher-dimensional and heterogeneous networks. In particular, analogous considerations apply to the family of “layered” networks described in note S7. This class consists of networks with arbitrary “baseline topology” made increasingly non-normal through a process of “directed stratification.” In addition, for these networks, one can define parameters α and ℓ that represent the directionality strength between adjacent layers and depth of connected layers, respectively. As in the chain network (Eq. 9), these parameters regulate departure from normality.

Mathematically normal versions of this chain architecture are obtained either when there effectively is no chain (set of isolated nodes) or when there is no specific directionality in the connectivity (α = 1, symmetric graph). In either case, the information rate decreases with increasing transmission window *T* (Fig. 3B, lowest curves), consistent with the formal theory developed above. To understand this behavior, and as a preliminary to our analysis of non-normal networks, we examine the optimal allocation of input power, or the spatial structure of the optimal input distribution. In Fig. 3C, we plot the optimal input covariance Σ^⋆^ (calculated as part of deriving the capacity; recall Eq. 5), expressed in the eigenbasis of the connectivity matrix ***A***, with eigenvectors sorted by decreasing values of their decay rate. For long transmission windows, more of the input variance is funneled through slow-decaying modes than through fast-decaying ones (right, *T* ≥ 0.5). This allows more of the input signal to survive the natural decay of activity in the network, thereby sustaining the SNR at the receiver. For shorter transmission windows, this strategy no longer pays off: Much of what is signal for the current transmission is effectively “noise” for the next transmission epoch, and prolonging its decay adds further intersymbol interference. Accordingly, the optimal allocation strategy for short *T* is the opposite of that for large *T*: Each subchannel is now allocated power proportional to its decay rate (note S5). Last, while achieving the information capacity requires careful selection of subchannels according to their decay rates (as just discussed), concentrating the input power on too few channels comes at a cost, as communication no longer exploits all the network’s degrees of freedom. This is best illustrated in a set of *n* independent nodes with identical time constants, for which the best strategy is provably to give each node an equal share *P*/*n* of the total available power (note S5). This amounts to maximizing the entropy of the input distribution. The covariances matrices of Fig. 3C represent the optimal way of resolving the above trade-offs for the chain architecture considered here.

We next show that large gains in information rate can be obtained by making the network connectivity non-normal. The degree of non-normality of the chain’s connectivity matrix (ℓ = 8) can be increased, without altering its eigenvalues, by increasing a single parameter α reflecting the graph’s directionality (Fig. 3A). As the network is made increasingly non-normal in this way, its information rate grows to eventually exceed the normal networks’ optimal rate by a large margin. Moreover, the optimal rate is now attained at some realistic, finite transmission window *T* (Fig. 3B, left).

To understand the mechanism through which non-normality improves information transmission, we repeat our inspection of optimal power allocation, now for a non-normal network with α = 7. In Fig. 3D, we plot the optimal input covariances (no longer expressed in the eigenbasis of ***A***, but in the standard basis of the network’s nodes) for various transmission window lengths. For large transmission windows, including the one that leads to the largest rate ℛ_max_, input power concentrates on the “source” nodes (leftmost nodes in Fig. 3A). This optimal strategy exploits the network’s ability to amplify signals as they propagate down the chain toward the “sink” (the last node). Thus, the SNR at the receiver can display large transient increases, whereas its decay could at best be slowed down in normal networks. For short transmission windows, such a strategy no longer pays off, because of the same trade-offs as uncovered above for normal networks. First, the signal transiently builds up into the next transmission epoch, where it no longer is signal but instead contributes noise. Second, unevenly distributing input power across the *n* network nodes by favoring the source nodes reduces the entropy of the input distribution, which fundamentally limits the information rate. Together, these drawbacks explain why the source nodes are not particularly favored over sink nodes when *T* is small (Fig. 3D, left), and why, in general, the input power does not concentrate entirely on the first node in the chain but is generally distributed among the first few.

To further substantiate that non-normality benefits the information capacity, we manipulate the degree of non-normality of the chain network discussed above, this time not by increasing α but through a complementary modification. Specifically, we morph the non-normal chain discussed above back into a normal network, by chopping the original chain of length ℓ = 8 into sets of shorter chains (Fig. 3A, top to bottom). Shorter chains consistently yield smaller information capacity (Fig. 3B, right), confirming that network non-normality has a positive impact on information transmission. We found a similar correlation for the more general class of layered topologies described in note S7. More precisely, for these networks, increasing the depth of connected layers has a provably beneficial effect on the communication performance.

### How noise shapes the optimal architecture

**Fig. 4:**
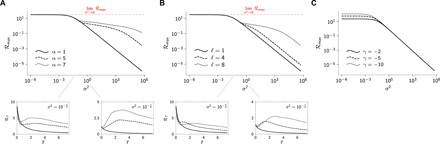
Maximum information rate versus noise level for chain network. (**A**) Plot of ℛ_max_ = max_*T*≥0_ ℛ*_T_* versus the output noise variance for the network of Fig. 3A with ***B*** = ***C*** = ***I***, γ = − 2.5, ℓ = 8, and three different values of α. (**B**) Plot of ℛ_max_ versus the output noise variance for the network of Fig. 3A with ***B*** = ***C*** = ***I***, γ = −2.5, α = 5, and three different values of ℓ. (**C**) Plot of ℛ_max_ versus the output noise variance for the network of Fig. 3A with ***B*** = ***C*** = ***I***, α = 1, ℓ = 8, and three different values of γ.

The results presented so far show that non-normal architectures can, in principle, outperform normal networks as information transmission media. These results were obtained for fixed input SNR, and we now show that non-normality is all the more beneficial as the SNR is poor. To show this, we revisit the chain architecture of the previous section (Fig. 3A) and systematically vary σ^2^, the amplitude of the noise at the receiver (Fig. 4).

In the low-noise regime, non-normality has little impact on information transmission, whether the network is made non-normal by increasing its directionality (Fig. 4A) or by increasing the length of its chains (Fig. 4B). For any ***A***, when σ^2^ is small, we have (cf. note S4)

$$R_{T}\approx -\frac{1}{\text{ln}\;2}\text{tr}\;(A)$$ (10)

which shows that, in the low-noise regime, the rate depends on the spectrum of ***A*** only. For the chain network, Eq. 10 reduces to $R_{T}\approx -\frac{\gamma n}{\text{ln}\;2}$, which is independent of α and ℓ. For large enough σ^2^, however, increasing α or ℓ has pronounced benefits on the maximum information rate ℛ_max_ (Fig. 4, A and B). In contrast, modifications of the parameters of the normal network (α = 1) that affect the eigenvalues without causing any departure from normality have close to no impact on the information rate. Specifically, changing the decay rate γ of the single nodes is only beneficial in the low-noise regime (Fig. 4C), corroborating the conclusions drawn from Eq. 8 above. The same equation also predicts that changing the overall coupling strength β (while keeping the directionality α constant) has no effect on ℛ_max_ (not shown).

From our analysis of this simple architecture, we conclude that network non-normality can greatly enhance information transmission in the low-SNR regime. We were able to show that non-normality can (in theory) cancel the effect of noise altogether (note S7). Specifically, it holds

$$\underset{\alpha \to \infty }{\text{lim}}R_{T}=-\frac{1}{\text{ln}\;2}\text{tr}\;(A)=-\frac{\gamma n}{\text{ln}\;2}$$ (11)

Equation 11 implies that, no matter how poor the SNR is, by increasing the degree of non-normality of the network via the directionality strength α, we get arbitrarily close to the maximum information rate achievable in the noiseless regime [by any network with identical value of tr(***A***); Fig. 4A, horizontal dashed red line].

Intriguingly, this result does not only hold for the simple line architecture described above, but also for a more complex class of “layered,” with the free parameter α summarizing departure from normality in terms of directionality strength between layers (note S7). In this family of models, as in the linear chain, the detrimental effect of output noise (however large) can be annihilated entirely by making the network sufficiently non-normal (by increasing α). In this limit of strong non-normality, the network effectively behaves as a one-dimensional channel with decay rate ∣tr(***A***)∣ and indeed achieves an information rate equal to that of any network with the same tr(***A***) in the absence of output noise (Eq. 10).

Last, we investigated how the noise level shapes the optimal architecture via an optimization approach. More precisely, we numerically computed the network architecture optimizing the maximum information rate ℛ_max_ with 10 nodes, bounded network weights, and different values of the noise variance σ^2^ (note S8). From our numerical analysis, it turns out that as σ^2^ grows, optimal networks become increasingly similar to a purely (hidden or effective) feedforward chain of maximal length, with approximately all of the input power allocated to the first nodes of the chain. This further corroborates our claim that non-normality is crucial for enhancing the communication performance of a network in the high-noise regime.

### Generalization to heterogeneous topologies

Although the formulae that we have derived regarding the information capacity of linear networks hold for arbitrary topology, most of the results presented so far were based either on highly simplified, small, and structured architectures (Fig. 3A) or on networks that deviated from normality in a highly structured way (note S7). To assess the generality of our results, we now study larger and more heterogeneous networks whose departure from normality we can also control. Specifically, we generate random connectivity matrices ***A*** following (*34*) as

A=(−I+S)P (12)

**Fig. 5:**
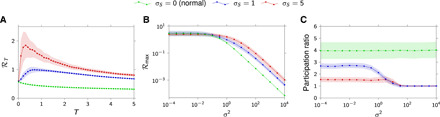
Role of non-normality in high-dimensional, heterogeneous networks. (**A**) Information rate as function of the transmission window *T* for *n* = 20, σ^2^ = 1, ***B*** = ***C*** = ***I***, and ***A*** drawn according to Eq. 12. (**B**) Maximum information rate ℛ_max_ = max_*T*≥0_ ℛ*_T_* as a function of the readout noise variance σ^2^. (**C**) Effective dimensionality [quantified using the “participation ratio”; see (*46*) and Materials and Methods] of the input distribution that defines the optimal allocation of input power as a function of the output noise variance σ^2^. In all panels, colors indicate the degree of non-normality (σ_s_) of the network. Light-colored regions denote 95% confidence interval (CI) around the mean, estimated from 50 independent realizations of the 20 × 20 random matrix ***A***, drawn according to Eq. 12.

Here, ***P*** is a random positive definite matrix drawn from the inverse Wishart distribution (see Materials and Methods), and ***S*** is a random skew-symmetric matrix whose (upper triangular) elements are drawn independently from a normal distribution with zero mean and variance $\sigma_{S}^{2}$. It is easily shown that any state matrix ***A*** drawn according to Eq. 12 implies stable network dynamics, despite the network graph showing apparent disorder with connections of arbitrary average magnitude (e.g., there is no limit to the norm of ***P*** and ***S***). The degree of network non-normality is set by the parameter σ*_S_*: When σ*_S_* = 0, ***A*** is symmetric, hence normal; as σ*_S_* increases, ***A*** departs further from normality (cf. see Materials and Methods and note S6). We calculated the maximum rate of these networks for various degrees of non-normality and found a similar interplay between network non-normality, transmission window, and input SNR as in the simplified architecture of Figs. 3 and 4. Specifically, non-normality results in greater maximum rates realized by nonzero optimal transmission windows (Fig. 5A). Moreover, these benefits over normal networks only arise in the low-SNR regime (Fig. 5B). Last, enhanced transmission performance at low SNR relies on a low-dimensional allocation of input power (Fig. 5C).

The role of non-normality in information transmission is further illuminated by considering the limit of poor SNR (σ^2^ → ∞): For any transmission window length *T* > 0, the rate decays with growing σ^2^ as (cf. note S4)

$$R_{T}\approx \frac{1}{2\text{ln}\;2\;T}\frac{∥B^{⊤}OB∥}{\sigma^{2}}$$ (13)

where $∥B^{⊤}OB∥$ represents the maximum total energy that the network can autonomously generate over a time window *T*, for an appropriate encoding of the input packet **u**_0_.

While the momentary magnitude of activity in normal networks can only decay in time (leading to sublinear growth of $∥B^{⊤}OB∥$ with *T*, i.e., decreasing ℛ*_T_* in Eq. 13), non-normal networks have the capacity to transiently amplify certain input codes before the eventual decay of signals implied by collective stability. This leads to superlinear growth of $∥B^{⊤}OB∥$ with *T*, which, in turn, results in transiently increasing ℛ*_T_* peaking at some finite value of *T* (Eq. 13).

Last, in deriving Eq. 13, we could also prove that in the limit of large noise σ^2^, the rate ℛ*_T_* is realized by effectively one-dimensional inputs, whose distribution lies entirely along the most sensitive input direction (i.e., along the initial condition that evokes the largest energy in the window *T*; note S4). In other words, the best way for the network to counteract a large amount of noise is to map every input packet onto a single, maximally amplified input pattern, thus effectively giving up on most of its degrees of freedom. This corroborates and strengthens the generality of our findings of Figs. 3D and 5C regarding the effective dimensionality of the input distribution in the high-noise regime.

## DISCUSSION

Here, we have proposed a novel framework to model information propagation through networks with arbitrary topology and nodes governed by linear dynamics. These dynamics imply a form of memory in single nodes, giving rise to interference between the activity transient initiated by the presentation of a given input packet and the activity left over from previous transmissions. We have used the notion of Shannon’s mutual information to quantify communication performance and study how the latter depends on the network architecture. Our analysis has shown that the qualitative effects of graph connectivity on communication are largely determined by a property that is often overlooked: the degree of non-normality of the network’s (weighted) adjacency matrix. In particular, we have shown that normal networks perform poorly in the presence of large readout noise at the receiver. In contrast, non-normal networks exhibit more favorable communication properties, including the ability to entirely cancel out the effect of readout noise provided that the input packets are appropriately encoded, and the adjacency matrix is sufficiently non-normal. Non-normal networks appear ubiquitous, with strong non-normality having been found in food webs, transport, and biological, social, communication, and citation networks (*22*). In addition, we mention that, besides information transfer, non-normality turns out to be the key to explaining and understanding a variety of other equally important phenomena, for instance, the process of pattern formation in natural and biological systems (*21*, *35*), the selective amplification of cortical activity patterns in the brain (*32*), and the emergence of giant oscillations in noise-driven dynamical systems (*36*–*38*).

**Fig. 6:**
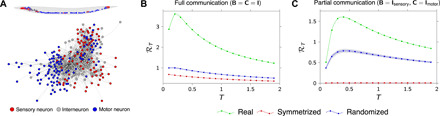
Information rate of *C. elegans* network. (**A**) Schematic of the chemical synapse network of the *C. elegans* nematode. The connectivity data and soma position of the neurons are taken from (*39*, *40*). (**B**) Plot of the information rate ℛ*_T_* versus transmission time *T* in the full communication setting (matrices ***B*** = ***C*** = ***I*** for the chemical synapse network ***A*** of the *C. elegans* (green curve), its symmetrized version (***A*** + ***A***^⊤^)/2 (red curve), and a randomized version in which each pair of entries (***A****_ij_*, ***A****_ji_*) has been swapped with probability 1/2 (blue curve). (**C**) Plot of the information rate ℛ*_T_* versus transmission time *T* in a partial communication setting (matrices ***B*** ≠ ***I*** and ***C*** ≠ ***I***). The green curve is the rate of the real network ***A***, with ***B*** and ***C*** selecting the 88 sensory and 107 motor neurons, respectively. The red curve is the rate of the symmetrized network described by matrix (***A*** + ***A***^⊤^)/2, with matrices ***B*** and ***C*** as above. The blue curve is the rate of the real network described by matrix ***A***, with matrices ***B*** and ***C*** randomly selecting 88 input nodes and 107 output nodes, respectively. The sets of input and output nodes are chosen to be nonoverlapping. In all plots, the networks have been stabilized, by shifting their spectrum by a scalar matrix γ***I***, γ ∈ ℝ, so that the real part of the largest eigenvalue is −0.1, and the variance of the output noise is set to σ^2^ = 1. In the randomized scenarios, the dash-dotted curves represent the mean over 100 realizations, and the light-blue regions denote 95% CI around the mean.

To further highlight the impact and potential practical relevance of our findings, we have used our framework to analyze the communication performance of the neuronal network of the nematode *Caenorhabditis elegans*. We focused on the (weighted and directed) chemical synapse network described in (*39*, *40*) and examined the linearized and stabilized network dynamics of the neuronal membrane potentials (see Materials and Methods). The network, which is illustrated in Fig. 6A, comprises 279 neurons (divided into 88 sensory neurons, 82 interneurons, and 107 motor neurons) recurrently coupled through 2194 inhibitory/excitatory synaptic connections. We first wondered whether the non-normality of this directed biological connectome had the beneficial impact on communication that we have documented here for artificial networks. We thus compared its information rate ℛ*_T_* (as a function of the transmission window *T*) with that of a symmetrized version (implying normal ***A***), as well as a randomized ensemble, wherein the direction of each existing coupling in the connectome is reversed with probability 1/2. Both manipulations induce a substantial drop in ℛ*_T_* from the real network (Fig. 6B), indicating that the *C. elegans* connectome is non-normal in a way that benefits information transmission as shown in this paper. We next wondered whether the network’s non-normal structure is likely to be exploited for communication by these organisms. We reasoned that communication would naturally flow from sensory neurons to motor neurons and that the network should therefore display good communication (in our framework) if, and only if, the input matrix ***B*** were to select sensory neurons while the output matrix ***C*** were to read out motor neurons. We found that this is indeed the case (compare Fig. 6C, green and blue). Notably, also, the symmetrized version of the connectome is almost unable to communicate information from sensory to motor nodes (Fig. 6B, red). Although preliminary, these numerical findings could shed light on the actual functioning of the *C. elegans* neuronal circuit and behavioral responsiveness to external stimuli. More generally, we expect that our theoretical framework could be used to understand and explain the emergence of certain topological structures in biological networks and to identify their intrinsic communication pathways.

In the paper, we focused on weighted networks and regarded the weights (and, precisely, their directionality and magnitude) as the main factors influencing network non-normality. However, non-normal architectures can also emerge in unweighted networks, e.g., in networks with heterogeneous outdegree/indegree distributions. It would therefore be interesting to investigate what the most relevant features affecting non-normality in unweighted networks are and to what extent these features affect communication performance. Further, in our framework, noise is modeled as the combined effect of readout noise and an internal, structured source of noise arising from intersymbol interference. Investigating how different noise models could affect our analysis and results represents a compelling direction of future research. Also, noise could play an active role in the information transfer process as the input source of the communication channel. This change of perspective could lead to an information-theoretic interpretation of the findings in (*36*–*38*), wherein non-normality has been linked with the emergence of amplified oscillations in noise-driven interconnected nonlinear systems.

As is well known in the theory of non-normal matrices and operators (*20*), strong departure from normality often implies heightened sensitivity to structural perturbations, for example, the random addition/deletion of nodes or edges in a graph. This suggests a generic trade-off between communication performance and resilience, which would be interesting to study further. For example, we note that in the low-noise regime, where normal networks can perform just as well as non-normal ones, constraints on robustness would favor normal networks. A similar trade-off has been identified recently in (*41*), where network resilience was shown to be generically at odds with network controllability.

Our work may also offer new perspectives on memory and information storage. Information transmission and storage are very similar problems: Communication is transmission through space, while memory is transmission through time. These two problems admit very similar models, are often both approached using the tools of information theory (*19*, *42*, *43*), and may interact in the context of network in ways that would be interesting to investigate further. Preliminary intuitions suggest that they may benefit each other: In our communication model, for example, intersymbol interference could be reduced if one could keep a memory of decoded past packets and subtract their individual contributions to the momentary network activity at any time. Conversely, communication may improve memory. An obvious example is the oral tradition in human communities, where transmission of information from generation to generation emerges as a way to overcome the finite memory span (and indeed, life span) of individuals.

## MATERIALS AND METHODS

### Gramian matrices and numerical computation of information capacity and rate

The observability Gramian over the interval [0, *T*] of the system in Eq. 1 is defined as

$$O=\int_{0}^{T}e^{A^{⊤}t}C^{⊤}{Ce}^{At}\;dt$$ (14)

and can be numerically evaluated via numerical integration of the matrix-valued differential equation

$$\dot{X}(t)=A^{⊤}X(t)+X(t)A+C^{⊤}C$$ (15)

subject to the initial condition ***X***(0) = 0 (*27*). The infinite-horizon controllability Gramian of the dynamics in Eq. 1 discretized with sampling time *T* as

$$W=\sum_{k=0}^{\infty }e^{A\mathrm{kT}}B\Sigma B^{⊤}e^{A^{⊤}\mathrm{kT}}$$ (16)

and can be computed as the solution of the discrete-time algebraic Lyapunov equation

$$X-e^{AT}{\mathrm{Xe}}^{A^{⊤}T}=B\Sigma B^{⊤}$$ (17)

In the numerical evaluation of the capacity and rate, the Gramians 14 and 16 have been computed via Eqs. 15 and 17, respectively. For vector-valued inputs (*m* ≥ 2), the solution of the optimization problem in Eqs. 5 and 6 has been numerically carried out in Python using optimization routines from the Pymanopt library (*44*), together with automatic differentiation techniques provided by Autograd (*45*). If ***B*** = ***C*** = ***I*** and *A* is normal, then the solution is unique and admits a closed-form expression in terms of eigenvalues of *A* (note S5). More generally, if ${\text{CC}}^{⊤}≽Ce^{A^{⊤}T}C^{⊤}Ce^{\mathrm{AT}}$, then the optimization in Eqs. 5 and 6 is convex (note S3), and so convergence to the maximum is always guaranteed using trust-region or steepest-descent methods. Otherwise, the problem turns out to be, in general, nonconvex, and to avoid local maxima, we ran the latter routines several times (10^2^ to 10^3^), starting from different random initializations, and selected the largest outcome.

### Generation of random non-normal matrices and participation ratio

In Eq. 12, the skew-symmetric matrix ***S*** ∈ ℝ^*n*×*n*^ has been generated as ***S*** = ***L*** − ***L***^⊤^, with $L_{\mathrm{ij}}∼N(0,\sigma_{S}^{2})$ for *i* < *j*, and ***L****_ij_* = 0 otherwise. The positive definite matrix ***P*** ∈ ℝ^*n*×*n*^ has been drawn from the inverse Wishart distribution with scale matrix ω^−2^***I*** and ν degrees of freedom. We chose ω^−2^ = ν − *n* − 1, ν = 24 + *n*, to guarantee sufficient heterogeneity in the eigenvalues of ***P*** (*34*). With this choice, it can be shown that $\sigma_{S}^{2}$ correlates well with standard measures of matrix non-normality (note S6). Following (*46*), given a positive definite matrix ***A*** ∈ ℝ^*n*×*n*^ with eigenvalues ${\left\{\lambda_{i}\right\}}_{i=1}^{n}$, we define the participation ratio

$$n_{\text{eff}}=\frac{{\left(\sum_{i=1}^{n}\lambda_{i}\right)}^{2}}{\sum_{i=1}^{n}\lambda_{i}^{2}}$$ (18)

When applied to the covariance matrix, the participation ratio provides a measure of the effective dimensionality of the underlying random vector.

### *C. elegans* dataset and network dynamics

The *C. elegans* connectivity data of (*39*, *40*) comprise two datasets: the gap junction and chemical synapse wiring diagrams. Since the gap junction dataset does not include link directionality, in our study, we focus on the chemical synapse network, which has clear directionality extracted from electron micrographs. This network consists of 279 neurons. These neurons are categorized in 88 sensory neurons (neurons known to respond to specific environmental conditions), 107 motor neurons (neurons characterized by the presence of neuromuscular junctions), and 82 interneurons (the remainder). The network comprises 2194 synaptic connections. As in (*39*), we make the common assumption that GABAergic neurons (26 neurons) make inhibitory synapses, whereas the rest of the neurons form excitatory synapses. We describe the autonomous dynamics of the chemical synapse network by the following linear system

$$\tau \dot{x}(t)=(A-\gamma I)x(t)$$ (19)

where *x* is the vector containing the membrane potentials of all neurons around an equilibrium, ***A*** is the adjacency matrix of the chemical synapse network, τ = ***C***/*g*, and γ = *g^m^*/*g*. Here, the parameters ***C***, *g*, and *g^m^* represent the (average) neuronal membrane capacitance, synaptic conductance, and membrane conductance, respectively [see (*39*, *47*) for further details]. In our numerical study, we set τ = 0.5 and tune γ to stabilize the network dynamics (Eq. 19). Specifically, we set the largest real part of the eigenvalues to −0.1. This yields γ ≈ 20, a value within the physiological range of *g^m^* and *g* (*48*). However, profiles of ℛ*_T_* qualitatively similar to those in Fig. 6 (A and B) have been obtained for a wide range of values of parameters γ < 0, τ > 0, and noise variance σ^2^.

## Supplementary Material

aba2282_SM.pdf

## SUPPLEMENTARY MATERIALS

Supplementary material for this article is available at http://advances.sciencemag.org/cgi/content/full/6/22/eaba2282/DC1
