# Supplementary material for: Efficient communication over complex dynamical networks: The role of matrix non-normality
Source: Sci Adv. 2020 May 27;6(22):eaba2282. doi: 10.1126/sciadv.aba2282 (PMC7253166; doi:10.1126/sciadv.aba2282)
Supplement: aba2282_SM.pdf [file aba2282_SM.pdf]

Science Advances
AAAS

advances.sciencemag.org/cgi/content/full/6/22/eaba2282/DC1

Supplementary Materials for

Efficient communication over complex dynamical networks: The role of matrix non-normality

Giacomo Baggio, Virginia Rutten, Guillaume Hennequin, Sandro Zampieri*

*Corresponding author. Email: sandro.zampieri@unipd.it

Published 27 May 2020, *Sci. Adv.* **6**, eaba2282 (2020)
DOI: 10.1126/sciadv.aba2282

This PDF file includes:

Notes S1 to S8
Figs. S1 to S5
References

1 Controllability and observability Gramians

Consider the continuous-time linear time-invariant system

$$\begin{aligned} \frac{\mathrm{d}x(t)}{\mathrm{d}t} &= Ax(t) + Bu(t), \\ y(t) &= Cx(t), \end{aligned} \tag{S1}$$

where $t \geq 0$ and $x(0) = x_0 \in \mathbb{R}^n$. Here, $x(t) \in \mathbb{R}^n$, $u(t) \in \mathbb{R}^m$, and $y(t) \in \mathbb{R}^p$ denote the state, input, and output of the system at time t, respectively, and A, B, and C are matrices of suitable dimensions. The controllability Gramian of the system over the interval $[0, T]$, $T > 0$, is defined as

$$\mathcal{W} = \int_0^T e^{At} BB^\top e^{A^\top t} \, \mathrm{d}t, \tag{S2}$$

whereas the observability Gramian of the system over the interval $[0, T]$, $T > 0$, as

$$\mathcal{O} = \int_0^T e^{A^\top t} C^\top C e^{At} \, \mathrm{d}t. \tag{S3}$$

The above-defined Gramians matrices $\mathcal{W}$ and $\mathcal{O}$ are always positive semidefinite, and they are related to the controllability and observability properties of the linear system in Equation S1. In particular, the system is controllable if and only if $\mathcal{W}$ is positive definite, and observable if and only if $\mathcal{O}$ is positive definite (see e.g. (*27*) for further details). Furthermore, if A is Hurwitz stable (that is, all the eigenvalues of A have negative real part), then $\mathcal{W}$ and $\mathcal{O}$ are well-defined for $T \to \infty$, and they correspond to the (unique) positive semidefinite solution of the continuous-time Lyapunov equations $AX + XA^\top = -BB^\top$ (controllability Gramian) and $A^\top X + XA = -C^\top C$ (observability Gramian). From an intuitive viewpoint, $\mathcal{W}$ is related to the energy needed to steer the system from an initial state x_0 to a desired target state x_f at time T, i.e. $x(T) = x_f$, whereas $\mathcal{O}$ to the energy of the system's free response evoked by a certain initial state x_0 in the interval $[0, T]$, see e.g. (*49, 50, 51*).

Similar definitions/interpretations hold for systems governed by discrete-time dynamics

$$\begin{aligned} x(k+1) &= Ax(k) + Bu(k), \\ y(k) &= Cx(k), \end{aligned} \tag{S4}$$

where $k \in \mathbb{N}_{>0}$ and $x(0) = x_0 \in \mathbb{R}^n$. In this case, the controllability and observability Gramians over the interval $[0, T]$, $T \in \mathbb{N}$, are given, respectively, by

$$\mathcal{W} = \sum_{k=0}^{T-1} A^k BB^\top (A^\top)^k \quad \text{and} \quad \mathcal{O} = \sum_{k=0}^{T-1} (A^\top)^k C^\top C A^k. \tag{S5}$$

If A is Schur stable (all the eigenvalues of A have modulus strictly less than one), then the infinite-horizon ($T \to \infty$) controllability and observability Gramians coincide with the positive semidefinite solution of the discrete-time Lyapunov equation $AXA^\top - X = -BB^\top$ and $A^\top XA - X = -C^\top C$, respectively.

2 Derivation of the information capacity formula

Here and throughout the paper, we indicate with $\det(X)$ and $\mathrm{tr}(X)$ the determinant and trace of matrix X, respectively, and $X \succcurlyeq 0$ ($X \succ 0$) means that X is positive semidefinite (positive definite, respectively). Further, given two continuous random variables x and y, we let $h(x)$ and $h(x|y)$ denote, respectively, the differential entropy of x and the conditional differential entropy of x given y. The mutual information between two continuous random variable x and y is given by $\mathcal{I}(x; y) = h(x) - h(x|y)$ (*26*).

We remark that the mutual information between the input and output messages of a communication channel, further optimized over all power-limited input distributions, coincides with the maximum achievable rate of reliable information transmission (Shannon's channel capacity) when the channel is *memoryless*, i.e., when past inputs do not affect the currently transmitted message (*26*). This is not, however, the case in our scenario because of inter-symbol interference. Moreover, even in the memoryless case, to achieve Shannon's capacity the encoder and the decoder must keep memory of previous transmissions. In most applications of network transmission (as those mentioned in the main text), instead, memory is not available, and so reconstruction has to be performed on a packet-by-packet basis.

To derive the capacity formula in Equation 5 of the main text, we first address the case $B = I$, and then we show how to extend the argument to the general case of rectangular B's. With reference to the communication channel described in the main text,

we focus, without any loss of generality, on the transmission delivered at time $t = 0$. The total information signal in the time window $[0, T]$ will be the superposition of the signals $y_k(t)$'s in the same window, namely $\sum_{k\in\mathbb{Z}} y_k(t)$, $0 \le t \le T$. In the latter summation, the signal that contains the "useful" information is given by $y_0(t)$, $0 \le t \le T$. Let $\mathcal{L}_2^p[0, T]$ be the Hilbert space of square integrable functions over the interval $[0, T]$ equipped with the inner product $\langle f, g\rangle_{\mathcal{L}_2} = \int_0^T f(t)g(t)\,\mathrm{d}t$, and note that $y_0(t)$, $0 \le t \le T$, belongs to the finite-dimensional subspace $\mathcal{Q}$ of $\mathcal{L}_2^p[0, T]$ generated by the functions $\{Ce^{At}e_i,\ t \in [0, T]\}_{i=1}^n$, where $\{e_i\}_{i=1}^n$ denotes the canonical basis vectors of $\mathbb{R}^n$. Thus, $y_0(t)$ can be written as

$$y_0(t) = \sum_{i=1}^{M} y_i f_i(t), \tag{S6}$$

where $\{f_i(t)\}_{i=1}^M$ is any orthonormal basis in $\mathcal{Q}$ and

$$y_i := \langle f_i(t), y_0(t)\rangle_{\mathcal{L}_2} = \int_0^T f_i^\top(t) y_0(t)\,\mathrm{d}t = \underbrace{\int_0^T f_i^\top(t) Ce^{At}\,\mathrm{d}t}_{=:\,F_i^\top}\, u_0. \tag{S7}$$

Let us define $F := [F_1, \dots, F_M]^\top$ and $Y_0 := [y_1, \dots, y_M]^\top$. For all $u_0 \in \mathbb{R}^n$, it holds

$$\langle y_0(t), y_0(t)\rangle_{\mathcal{L}_2} = u_0^\top \int_0^T e^{A^\top t} C^\top C e^{At}\,\mathrm{d}t\, u_0 = Y_0^\top Y_0 = u_0^\top F^\top F u_0. \tag{S8}$$

The latter equation implies that $F^\top F = \mathcal{O}$ where $\mathcal{O} = \int_0^T e^{A^\top t} C^\top C e^{At}\,\mathrm{d}t$ is the observability Gramian of the pair (A, C) over the interval $[0, T]$. The covariance between two components y_h and y_ℓ, $h, \ell = 1, 2, \dots, M$, is given by $\mathbb{E}[y_h y_\ell] = \mathbb{E}\left[F_h^\top u_0 u_0^\top F_\ell\right] = F_h^\top \Sigma F_\ell$, where $\Sigma := \mathbb{E}[u_0 u_0^\top]$. From this fact, it follows that the covariance of the useful signal $y_0(t)$ is

$$\Sigma_{y_0} := \mathbb{E}\left[Y_0 Y_0^\top\right] = F\Sigma F^\top. \tag{S9}$$

The overall channel noise, here denoted by $r(t)$, is modelled as the sum of two contributions, namely:

(i) the additive Gaussian white noise $n(t)$, and

(ii) the interference term $i(t)$ due to inter-symbol interference.

The noise term $r(t) = n(t) + i(t)$ can be written as, w.r.t. the previously introduced orthonormal basis $\{f_i(t)\}_{i=1}^M$ of $\mathcal{Q}$,

$$r(t) = \sum_{i=1}^{M} r_i f_i(t) + r_\perp(t), \tag{S10}$$

where $r_\perp(t)$ belongs to the orthogonal complement of $\mathcal{Q}$ and

$$r_i := \langle f_i(t), r(t)\rangle_{\mathcal{L}_2} = \int_0^T f_i^\top(t)\left(Ce^{At}\sum_{k=1}^{\infty} e^{AkT} u_{-k} + n(t)\right)\mathrm{d}t = F_i^\top \sum_{k=1}^{\infty} e^{AkT} u_{-k} + n_i, \tag{S11}$$

with $n_i := \int_0^T f_i(t)^\top n(t)\,\mathrm{d}t$. The covariance between two noise components r_h, r_ℓ, $h, \ell = 1, 2, \dots, M$, is given by

$$\mathbb{E}[r_h r_\ell] = F_h^\top \sum_{k=1}^{\infty} e^{AkT}\mathbb{E}[u_{-k}u_{-k}^\top] e^{A^\top kT} F_\ell + \sigma^2 \int_0^T\int_0^T f_h^\top(t) f_\ell(\tau)\delta(t-\tau)\,\mathrm{d}t\,\mathrm{d}\tau = F_h^\top \sum_{k=1}^{\infty} e^{AkT}\Sigma e^{A^\top kT} F_\ell + \sigma^2\delta_{h,\ell}, \tag{S12}$$

where $\delta_{h,\ell}$ denotes the Kronecker delta function and we implicitly used the fact that $\{u_k\}$ are i.i.d. random variables, and u_k and n_i are independent for all k and i. Therefore, by defining $R := [r_1, \dots, r_M]^\top$, we have that the covariance of the noise component in $\mathcal{Q}$ is given by

$$\Sigma_r := \mathbb{E}\left[RR^\top\right] = F\sum_{k=1}^{\infty} e^{AkT}\Sigma e^{A^\top kT} F^\top + \sigma^2 I = F(\mathcal{W} - \Sigma)F^\top + \sigma^2 I, \tag{S13}$$

where $\mathcal{W} := \sum_{k=0}^{\infty} e^{AkT}\Sigma e^{A^\top kT}$ is the infinite-horizon controllability Gramian of the discretized pair $(e^{AT}, \Sigma^{1/2})$.

By exploiting the above finite-dimensional representation of the output signal and noise term and the covariance expressions in Equations S9 and S13, we can now compute the mutual information (as measured in bits) between the channel input u_0 and the corrupted output trajectory over the window $[0, T]$, $\tilde{Y}_0$,

$$\begin{aligned}\mathcal{I}_T(u_0, \tilde{Y}_0) &= h(\tilde{Y}_0) - h(\tilde{Y}_0|u_0) = h(Y_0 + R) - h(Y_0 + R|u_0) = h(Y_0 + R) - h(R) \\ &= \frac{1}{2}\log_2 \frac{\det(\Sigma_{y_0} + \Sigma_r)}{\det \Sigma_r} = \frac{1}{2}\log_2 \frac{\det(\sigma^2 I + \mathcal{O}\mathcal{W})}{\det(\sigma^2 I + \mathcal{O}(\mathcal{W} - \Sigma))},\end{aligned} \tag{S14}$$

where we used the properties of the differential entropy, the fact that Y_0 and R are independent Gaussian random variables (*26, Ch. 8*), and, in the last step, the similarity invariance of the determinant and the fact that $F^\top F = \mathcal{O}$. Finally, the previous expression yields the expression of the information capacity with power constraint P,

$$\mathcal{C}_T = \frac{1}{2} \max_{\Sigma \succcurlyeq 0, \mathrm{tr}\,\Sigma \leq P} \mathcal{I}_T(u_0, \tilde{Y}_0) = \frac{1}{2} \max_{\Sigma \succcurlyeq 0, \mathrm{tr}\,\Sigma \leq P} \log_2 \frac{\det(\sigma^2 I + \mathcal{O}\mathcal{W})}{\det(\sigma^2 I + \mathcal{O}(\mathcal{W} - \Sigma))}. \tag{S15}$$

This concludes the proof for the case $B = I$.

For general input matrices $B \in \mathbb{R}^{n\times m}$, the search space $\{\Sigma \in \mathbb{R}^{n\times n},\ \Sigma \succcurlyeq 0,\ \mathrm{tr}\ \Sigma \leq P\}$ in the maximization of the previously derived formula should be replaced by $\{B\Sigma B^\top\ :\ \Sigma \in \mathbb{R}^{m\times m},\ \Sigma \succcurlyeq 0,\ \mathrm{tr}\ \Sigma \leq P\}$. Hence, it holds

$$\mathcal{C}_T = \frac{1}{2} \max_{\Sigma \succcurlyeq 0, \mathrm{tr}\ \Sigma \leq P} \log_2 \frac{\det(\sigma^2 I + \mathcal{O}\mathcal{W})}{\det(\sigma^2 I + \mathcal{O}(\mathcal{W} - B\Sigma B^\top))}, \tag{S16}$$

where $\mathcal{W} := \sum_{k=0}^{\infty} e^{AkT} B\Sigma B^\top e^{A^\top kT}$ is the infinite-horizon controllability Gramian of the discretized pair $(e^{AT}, B\Sigma^{1/2})$.

To conclude, we show that the constraint $\mathrm{tr}\ \Sigma \leq P$ in Equation S16 can be replaced by $\mathrm{tr}\ \Sigma = P$. To this end, Let us define

$$f_T(\Sigma) := \log_2 \frac{\det(\sigma^2 I + \mathcal{O}\mathcal{W})}{\det(\sigma^2 I + \mathcal{O}(\mathcal{W} - B\Sigma B^\top))}. \tag{S17}$$

We will show that $f_T(\alpha\Sigma)$ is a monotonically increasing function of $\alpha \geq 0$, that is, for any $\alpha_1,\ \alpha_2 \geq 0$ such that $\alpha_1 \leq \alpha_2$, it holds $f_T(\alpha_1\Sigma) \leq f_T(\alpha_2\Sigma)$. The latter fact clearly implies that the optimal Σ maximizing $f_T(\Sigma)$ under the constraint $\mathrm{tr}\ \Sigma \leq P$, must satisfy the latter constraint with equality. We have

$$\begin{aligned}f_T(\alpha\Sigma) &= \log_2 \frac{\det(\sigma^2 I + \alpha\mathcal{O}\mathcal{W})}{\det(\sigma^2 I \alpha\mathcal{O}(\mathcal{W} - B\Sigma B^\top))} \\ &= \log_2 \frac{\det(\alpha\mathcal{O}^{1/2}\mathcal{W}\mathcal{O}^{1/2} + \sigma^2 I)}{\det(\alpha\mathcal{O}^{1/2}(\mathcal{W} - B\Sigma B^\top)\mathcal{O}^{1/2} + \sigma^2 I)} \\ &= \sum_{i=1}^{N} \log_2(\alpha\lambda_i + \sigma^2) - \sum_{i=1}^{N} \log_2(\alpha\mu_i + \sigma_r^2),\end{aligned} \tag{S18}$$

where $\{\lambda_i\}_{i=1}^n$ and $\{\mu_i\}_{i=1}^n$ denotes the ordered eigenvalues of $\mathcal{O}^{1/2}\mathcal{W}\mathcal{O}^{1/2}$ and $\mathcal{O}^{1/2}(\mathcal{W} - B\Sigma B^\top)\mathcal{O}^{1/2}$, respectively. Taking the derivative w.r.t. α of the previous expression, we get

$$\frac{\mathrm{d}f_T(\alpha\Sigma)}{\mathrm{d}\alpha} = \frac{1}{\ln 2}\sum_{i=1}^{N} \frac{\lambda_i}{\alpha\lambda_i + \sigma^2} - \frac{1}{\ln 2}\sum_{i=1}^{N} \frac{\mu_i}{\alpha\mu_i + \sigma^2}. \tag{S19}$$

Since $\mathcal{O}^{1/2}\mathcal{W}\mathcal{O}^{1/2} \succcurlyeq \mathcal{O}^{1/2}(\mathcal{W} - B\Sigma B^\top)\mathcal{O}^{1/2}$, then $\lambda_i \geq \mu_i$, $i = 1, 2, \dots, n$. This yields

$$\frac{\mathrm{d}f_T(\alpha\Sigma)}{\mathrm{d}\alpha} = \frac{1}{\ln 2}\sum_{i=1}^{N} \frac{\lambda_i - \mu_i}{(\lambda_i\alpha + \sigma^2)(\mu_i\alpha + \sigma^2)} \geq 0, \tag{S20}$$

which, in turn, implies that $f_T(\alpha\Sigma)$ is a monotonically increasing function of $\alpha \geq 0$, as required.

3 Properties of the information capacity and rate

In this Supplementary Note, we collect and discuss some key properties of the information capacity $\mathcal{C}_T$ and information rate $\mathcal{R}_T$. The first result asserts that $\mathcal{C}_T$ (and, therefore, $\mathcal{R}_T$) does not independently depend on the available power P and readout noise variance σ^2, but on the "signal-to-noise" ratio P/σ^2 only.

Proposition 1 (Scaling invariance of $\mathcal{C}_T$ w.r.t. P and σ^2). *For all $\alpha > 0$, it holds*

$$\mathcal{C}_T(P, \sigma^2) = \mathcal{C}_T(\alpha P, \alpha\sigma^2), \tag{S21}$$

where we made explicit the dependence of $\mathcal{C}_T$ on P and σ^2.

Proof. First observe that if we replace Σ and σ^2 with $\alpha\Sigma$ and $\alpha\sigma^2$, respectively, then $\mathcal{W}$ is replaced with $\alpha\mathcal{W}$. This in turn implies that the value of

$$f_T(\Sigma, \sigma^2) := \log_2 \frac{\det(\sigma^2 I + \mathcal{O}\mathcal{W})}{\det(\sigma^2 I + \mathcal{O}(\mathcal{W} - B\Sigma B^\top))} \tag{S22}$$

is not affected by this change of variables. If $\Sigma^\star$ is the input covariance maximizing $f_T(\Sigma, \sigma^2)$ under the constraint $\operatorname{tr}\Sigma = P$, it holds

$$\mathcal{C}_T(\alpha P, \alpha\sigma^2) \geq f_T(\alpha\Sigma^\star, \alpha\sigma^2) = f_T(\Sigma^\star, \sigma^2) = \mathcal{C}_T(P, \sigma^2). \tag{S23}$$

On the other hand, let $\Sigma^\star \succcurlyeq 0$ now denote the input covariance maximizing $f_T(\Sigma, \alpha\sigma^2)$ under the constraint $\operatorname{tr}\Sigma = \alpha P$. It holds

$$\mathcal{C}_T(P, \sigma^2) \geq f_T\left(\frac{1}{\alpha}\Sigma^\star, \sigma^2\right) = f_T(\Sigma^\star, \alpha\sigma^2) = \mathcal{C}_T(\alpha P, \alpha\sigma^2). \tag{S24}$$

Therefore, it must be $\mathcal{C}_T(\alpha P, \alpha\sigma^2) = \mathcal{C}_T(P, \sigma^2)$. □

We now investigate the convexity properties of the maximizing function in Equation S16, namely

$$f_{T,\sigma^2}(\Sigma) := \frac{1}{2}\log_2 \frac{\det\left(\sigma^2 I + \mathcal{O}\mathcal{W}\right)}{\det\left(\sigma^2 I + \mathcal{O}(\mathcal{W} - B\Sigma B^\top)\right)}, \tag{S25}$$

where we used the subscripts T and σ^2 to make explicit the dependence of f on the transmission time window and output noise variance, respectively. The following result provides a sufficient condition under which the $f_{T,\sigma^2}(\Sigma)$ is a concave function of $\Sigma \succcurlyeq 0$.

Proposition 2 (Concavity of $f_{T,\sigma^2}(\Sigma)$). *Assume that the pair (A, C) is observable. If the following condition is satisfied*

$$C^\top C \succcurlyeq e^{A^\top T} C^\top C e^{AT}, \tag{S26}$$

then $f_{T,\sigma^2}(\Sigma)$ is a concave function of $\Sigma \succcurlyeq 0$.

Proof. Since (A, C) is observable, $\mathcal{O}$ is positive definite, and hence invertible. This implies that $\mathcal{W} + \sigma^2\mathcal{O}^{-1}$ is positive definite for every $\Sigma \succcurlyeq 0$. After some algebraic manipulations, we can rewrite $f_{T,\sigma^2}(\Sigma)$ as

$$\begin{aligned}
f_{T,\sigma^2}(\Sigma) &= \frac{1}{2}\log_2 \frac{\det(\sigma^2\mathcal{O}^{-1} + \mathcal{W})}{\det(\sigma^2\mathcal{O}^{-1} + e^{AT}\mathcal{W}e^{A^\top T})} \\
&= \frac{1}{2}\log_2 \frac{\det(\sigma^2\mathcal{O}^{-1} + \mathcal{W})}{\det e^{2AT}\det(\sigma^2 e^{-AT}\mathcal{O}^{-1}e^{-A^\top T} + \mathcal{W})} \\
&= \frac{1}{2}\log_2\det\left[(\sigma^2\mathcal{O}^{-1} + \mathcal{W})(\sigma^2 e^{-AT}\mathcal{O}^{-1}e^{-A^\top T} + \mathcal{W})^{-1}\right] + \frac{1}{2}\log_2\det e^{-2AT} \\
&= -\frac{1}{2}\log_2\det\left[(\sigma^2\mathcal{O}^{-1} + \mathcal{W})^{-1}(\sigma^2 e^{-AT}\mathcal{O}^{-1}e^{-A^\top T} + \mathcal{W})\right] + \frac{1}{2}\log_2\det e^{-2AT} \\
&= -\frac{1}{2\ln 2}\ln\det\left(I + X^{-1}K\right) - \frac{T}{\ln 2}\operatorname{tr}(A),
\end{aligned} \tag{S27}$$

where we have defined $X := \sigma^2\mathcal{O}^{-1} + \mathcal{W}$ and $K := \sigma^2 e^{-AT}\mathcal{O}^{-1}e^{-A^\top T} - \sigma^2\mathcal{O}^{-1}$. Next, since

(i) $X \succ 0$ is a linear function of Σ, and

(ii) from (*52*), $\ln\det\left(I + X^{-1}K\right)$ is a convex function of X if $K \succcurlyeq 0$,

it follows that $f_{T,\sigma^2}(\Sigma)$ is concave if $K \succcurlyeq 0$. To conclude we notice that the latter condition is satisfied if

$$\begin{aligned} K \succcurlyeq 0 \;&\Leftrightarrow\; \sigma^2 e^{-AT}\mathcal{O}^{-1}e^{-A^\top T} - \sigma^2\mathcal{O}^{-1} \succcurlyeq 0 \\ &\Leftrightarrow\; e^{-AT}\mathcal{O}^{-1}e^{-A^\top T} - \mathcal{O}^{-1} \succcurlyeq 0 \\ &\Leftrightarrow\; -e^{A^\top T}\mathcal{O}e^{AT} + \mathcal{O} \succcurlyeq 0 \\ &\Leftrightarrow\; \int_0^T e^{A^\top t}(-e^{A^\top T}C^\top Ce^{AT} + C^\top C)e^{AT}\mathrm{d}t \succcurlyeq 0 \\ &\Leftrightarrow\; -e^{A^\top T}C^\top Ce^{AT} + C^\top C \succcurlyeq 0. \end{aligned} \tag{S28}$$

This completes the proof. □

As a side remark, it is worth pointing out that the condition in Proposition 2 always holds if A is stable and normal and $C = I$, since $\|e^X\| = \lambda_{\max}(e^X(e^X)^\top) < 1$ for normal and stable X's, where $\lambda_{\max}(\cdot)$ denotes the largest eigenvalue of a symmetric matrix.

Next, we focus on the monotonicity properties of $f_{T,\sigma^2}(\Sigma)$ with respect to σ^2.

Proposition 3 (Monotonicity of $f_{T,\sigma^2}(\Sigma)$ w.r.t. σ^2). *For all $\sigma_2^2 \geq \sigma_1^2 > 0$ and $\Sigma \succcurlyeq 0$, it holds*

$$f_{T,\sigma_1^2}(\Sigma) \geq f_{T,\sigma_2^2}(\Sigma), \tag{S29}$$

$$\sigma_1^2 f_{T,\sigma_1^2}(\Sigma) \leq \sigma_2^2 f_{T,\sigma_2^2}(\Sigma). \tag{S30}$$

Proof. Given $\sigma^2 > 0$, by using the properties of logarithms, $f_{T,\sigma^2}(\Sigma)$ can be rewritten as

$$f_{T,\sigma^2}(\Sigma) = \sum_{i=1}^n \log_2\left(\frac{1+\lambda_i/\sigma^2}{1+\mu_i/\sigma^2}\right), \tag{S31}$$

where $\{\lambda_i\}_{i=1}^n$, $\lambda_1 \geq \cdots \geq \lambda_n$, and $\{\mu_i\}_{i=1}^n$, $\mu_1 \geq \cdots \geq \mu_n$, are the eigenvalues of the positive (semi)definite matrices $\mathcal{O}^{1/2}\mathcal{W}\mathcal{O}^{1/2}$ and $\mathcal{O}^{1/2}(\mathcal{W} - B\Sigma B^\top)\mathcal{O}^{1/2}$, respectively. By virtue of Weyl's Monotonicity Theorem (*53, Corollary III.2.3*), it holds $\lambda_i \geq \mu_i$ for all $i = 1, 2, \ldots, n$. It is now a matter of direct computation to show that if $\lambda > \mu > 0$,

$$\frac{\mathrm{d}}{\mathrm{d}x}\log_2\left(\frac{1+\lambda/x}{1+\mu/x}\right) > 0, \quad x > 0, \tag{S32}$$

$$\frac{\mathrm{d}}{\mathrm{d}x}x\log_2\left(\frac{1+\lambda/x}{1+\mu/x}\right) < 0, \quad x > 0. \tag{S33}$$

In view of the latter inequalities, it follows that each term in the sum defining $f_{T,\sigma^2}(\Sigma)$ is a monotonically decreasing function of $\sigma^2 > 0$, whereas each term in the sum defining $\sigma^2 f_{T,\sigma^2}(\Sigma)$ is monotonically increasing function of $\sigma^2 > 0$. □

Proposition 4 (Monotonicity of $f_{T,\sigma^2}(\Sigma)$ w.r.t. T). *Suppose that the pair (A, C) is observable and $\sigma^2 > 0$. For all $T_1 > 0$, $T_2 = hT_1$, $h \in \mathbb{N}_{>0}$, and $\Sigma \succcurlyeq 0$, it holds*

$$f_{T_2,\sigma^2}(\Sigma) \geq f_{T_1,\sigma^2}(\Sigma). \tag{S34}$$

Proof. Since $T_2 \geq T_1$, we have

$$\mathcal{O}_{T_2} := \int_0^{T_2} e^{A^\top t}C^\top Ce^{At}\mathrm{d}t \succcurlyeq \int_0^{T_1} e^{A^\top t}C^\top Ce^{At}\mathrm{d}t =: \mathcal{O}_{T_1} \succ 0, \tag{S35}$$

where positive definiteness follows from observability of the pair (A, C). Furthermore, since $T_2 = hT_1$, $h \in \mathbb{N}_{>0}$,

$$\mathcal{W}_{T_1} := \sum_{k=0}^\infty e^{AkT_1}B\Sigma B^\top e^{A^\top kT_1} \succcurlyeq \sum_{k=0}^\infty e^{AkhT_1}B\Sigma B^\top e^{A^\top khT_1} =: \mathcal{W}_{T_2}. \tag{S36}$$

Next, we can rewrite $f_{T,\sigma^2}(\Sigma)$ as

$$\begin{aligned} f_{T,\sigma^2}(\Sigma) &= \frac{1}{2}\log_2 \det\left[\left(\sigma^2 I + \mathcal{O}\mathcal{W}\right)\left(\sigma^2 I + \mathcal{O}(\mathcal{W} - B\Sigma B^\top)\right)^{-1}\right] \\ &= \frac{1}{2}\log_2 \det\left[I + \mathcal{O}B\Sigma B^\top\left(\sigma^2 I + \mathcal{O}(\mathcal{W} - B\Sigma B^\top)\right)^{-1}\right] \\ &= \frac{1}{2}\log_2 \det\left[I + B\Sigma B^\top\left(\sigma^2 I + \mathcal{O}(\mathcal{W} - B\Sigma B^\top)\right)^{-1}\mathcal{O}\right] \\ &= \frac{1}{2}\log_2 \det\left[I + B\Sigma B^\top((\mathcal{W} - B\Sigma B^\top) + \sigma^2\mathcal{O}^{-1})^{-1}\right] \\ &= \frac{1}{2}\log_2 \det\left[I + (B\Sigma B^\top)^{1/2}(e^{AT}\mathcal{W}e^{A^\top T} + \sigma^2\mathcal{O}^{-1})^{-1}(B\Sigma B^\top)^{1/2}\right], \end{aligned} \tag{S37}$$

In view of Equations S35 and S36, we have $\mathcal{O}_{T_2}^{-1} \preccurlyeq \mathcal{O}_{T_1}^{-1}$, and

$$\begin{aligned} & \mathcal{W}_{T_1} + \sigma^2\mathcal{O}_{T_1}^{-1} \succcurlyeq \mathcal{W}_{T_2} + \sigma^2\mathcal{O}_{T_2}^{-1} \\ \Rightarrow\ & \left(\mathcal{W}_{T_1} + \sigma^2\mathcal{O}_{T_1}^{-1}\right)^{-1} \preccurlyeq \left(\mathcal{W}_{T_2} + \sigma^2\mathcal{O}_{T_2}^{-1}\right)^{-1} \\ \Rightarrow\ & (B\Sigma B^\top)^{1/2}\left(\mathcal{W}_{T_1} + \sigma^2\mathcal{O}_{T_1}^{-1}\right)^{-1}(B\Sigma B^\top)^{1/2} \preccurlyeq (B\Sigma B^\top)^{1/2}\left(\mathcal{W}_{T_2} + \sigma^2\mathcal{O}_{T_2}^{-1}\right)^{-1}(B\Sigma B^\top)^{1/2} \\ \Rightarrow\ & I + (B\Sigma B^\top)^{1/2}\left(\mathcal{W}_{T_1} + \sigma^2\mathcal{O}_{T_1}^{-1}\right)^{-1}(B\Sigma B^\top)^{1/2} \preccurlyeq I + (B\Sigma B^\top)^{1/2}\left(\mathcal{W}_{T_2} + \sigma^2\mathcal{O}_{T_2}^{-1}\right)^{-1}(B\Sigma B^\top)^{1/2}. \end{aligned} \tag{S38}$$

Finally, from Equation S37, the latter inequality implies $f_{T_2,\sigma^2}(\Sigma) \geq f_{T_1,\sigma^2}(\Sigma)$. □

We note that, in the limit $T \to \infty$, $\mathcal{W} \to B\Sigma B^\top$ and $\mathcal{O} \to \int_0^T e^{A^\top t}C^\top Ce^{At}\,\mathrm{d}t = \mathcal{O}_\infty$, which are both finite matrices since A is stable. Hence, it follows that

$$\lim_{T\to\infty} f_{T,\sigma^2}(\Sigma) = \log_2 \det\left(I + \frac{1}{\sigma^2}\mathcal{O}_\infty B\Sigma B^\top\right), \tag{S39}$$

which is finite. From Proposition 4, this in turn implies that $f_{T,\sigma^2}(\Sigma)$ attains its maximum for $T \to \infty$.

To conclude, we establish a monotonicity property of the capacity with respect to a particular choice of the input and output matrices B and C.

Proposition 5 (Monotonicity of $\mathcal{C}_T$ w.r.t. B and C). *Assume that the pair (A, C) is observable, and let*

$$B_1 = \begin{bmatrix} I_{m_1} \\ 0 \end{bmatrix}, \quad C_1 = \begin{bmatrix} 0 & I_{p_1} \end{bmatrix}, \tag{S40}$$

$$B_2 = \begin{bmatrix} I_{m_2} \\ 0 \end{bmatrix}, \quad C_2 = \begin{bmatrix} 0 & I_{p_2} \end{bmatrix}, \tag{S41}$$

be two pairs of input and output matrices. If $m_2 \geq m_1$ and $p_2 \geq p_1$, then

$$\mathcal{C}_T(B_1, C_1) \leq \mathcal{C}_T(B_2, C_2), \tag{S42}$$

where we made explicit the dependence of $\mathcal{C}_T$ on the input matrix B and output matrix C.

Proof. First, as in the proof of Proposition 4, we have that Equation S25 can be rewritten as

$$f_{T,\sigma^2}(\Sigma) = \frac{1}{2}\log_2 \det\left[I + (B\Sigma B^\top)^{1/2}(e^{AT}\mathcal{W}e^{A^\top T} + \sigma^2\mathcal{O}^{-1})^{-1}(B\Sigma B^\top)^{1/2}\right], \tag{S43}$$

Next, since $p_2 \geq p_1$, we have

$$\mathcal{O}_1 := \int_0^T e^{A^\top t}C_1^\top C_1 e^{At}\,\mathrm{d}t \preccurlyeq \int_0^T e^{A^\top t}C_2^\top C_2 e^{At}\,\mathrm{d}t =: \mathcal{O}_2. \tag{S44}$$

From the previous inequality and the observability of (A, C), we have $\mathcal{O}_1^{-1} \succcurlyeq \mathcal{O}_2^{-1}$. This in turn implies that

$$\begin{aligned} & \mathcal{W} + \sigma^2\mathcal{O}_1^{-1} \succcurlyeq \mathcal{W} + \sigma^2\mathcal{O}_2^{-1} \\ \Rightarrow\ & \left(\mathcal{W} + \sigma^2\mathcal{O}_1^{-1}\right)^{-1} \preccurlyeq \left(\mathcal{W} + \sigma^2\mathcal{O}_2^{-1}\right)^{-1} \\ \Rightarrow\ & (B\Sigma B^\top)^{1/2}\left(\mathcal{W} + \sigma^2\mathcal{O}_1^{-1}\right)^{-1}(B\Sigma B^\top)^{1/2} \preccurlyeq (B\Sigma B^\top)^{1/2}\left(\mathcal{W} + \sigma^2\mathcal{O}_2^{-1}\right)^{-1}(B\Sigma B^\top)^{1/2} \\ \Rightarrow\ & I - (B\Sigma B^\top)^{1/2}\left(\mathcal{W} + \sigma^2\mathcal{O}_1^{-1}\right)^{-1}(B\Sigma B^\top)^{1/2} \preccurlyeq I - (B\Sigma B^\top)^{1/2}\left(\mathcal{W} + \sigma^2\mathcal{O}_2^{-1}\right)^{-1}(B\Sigma B^\top)^{1/2} \end{aligned} \tag{S45}$$

which in view of Equation S37 yields

$$f_{T,\sigma^2}(\Sigma, B, C_2) \geq f_{T,\sigma^2}(\Sigma, B, C_1), \tag{S46}$$

for any $B \in \mathbb{R}^{n\times m}$ and $\Sigma \succcurlyeq 0$, where we made explicit the dependence of $f_{T,\sigma^2}(\cdot)$ on the input matrix B and output matrix C. From the previous inequality, it follows that

$$\mathcal{C}_T(B, C_1) = \max_{\Sigma\succcurlyeq 0, \text{tr}\,\Sigma = P} f_{T,\sigma^2}(\Sigma, B, C_1) \leq \max_{\Sigma\succcurlyeq 0, \text{tr}\,\Sigma = P} f_{T,\sigma^2}(\Sigma, B, C_2) = \mathcal{C}_T(B, C_2). \tag{S47}$$

Now, let us define

$$\mathcal{S}_1 := \{B_1\Sigma_1 B_1 \,:\, \Sigma_1 \succcurlyeq 0,\ \text{tr}\ \Sigma_1 = P\}, \quad \mathcal{S}_2 := \{B_2\Sigma_2 B_2 \,:\, \Sigma_2 \succcurlyeq 0,\ \text{tr}\ \Sigma_2 = P\}. \tag{S48}$$

Since $m_2 \geq m_1$, we have $\mathcal{S}_1 \subseteq \mathcal{S}_2$. This in turn implies that

$$\mathcal{C}_T(B_1, C) = \max_{\Sigma\succcurlyeq 0, \text{tr}\,\Sigma = P} f_{T,\sigma^2}(\Sigma, B_1, C) = \max_{\Sigma\in\mathcal{S}_1} f_{T,\sigma^2}(\Sigma, I, C) \leq \max_{\Sigma\in\mathcal{S}_2} f_{T,\sigma^2}(\Sigma, I, C) = \max_{\Sigma\succcurlyeq 0, \text{tr}\,\Sigma = P} f_{T,\sigma^2}(\Sigma, B_2, C) = \mathcal{C}_T(B_2, C). \tag{S49}$$

Finally, a combination of Equations S47 and S49 yields the thesis. □

Proposition 5 in particular implies that the maximum capacity is always attained by picking $B = C = I$, that is, by selecting all nodes in the network as input/output nodes.

4 Information rate in the low and high noise regime

Here, we analyze the behavior of the information rate $\mathcal{R}_T$ in the limit cases where σ^2 tends to either zero or infinity.

Theorem 1 (Behavior of $\mathcal{R}_T$ in the low noise regime). *Let $A \in \mathbb{R}^{n\times n}$ be a stable matrix and assume that the pair (A, C) is observable. For all $T > 0$, it holds*

$$\lim_{\sigma^2\to 0} \mathcal{R}_T = -\frac{1}{\ln 2}\,\text{tr}(A). \tag{S50}$$

Further, as $\sigma^2 \to 0$, the optimal Σ in Equation S16 is any positive semidefinite unit-trace matrix such that $(A, B\Sigma^{1/2})$ is controllable.

Proof. Consider $f_{T,\sigma^2}(\Sigma)$ as defined in Equation S25. Since $f_{T,\sigma^2}(\Sigma)$ and $\max_{\Sigma\succcurlyeq 0, \text{tr}\,\Sigma=1} f_{T,\sigma^2}(\Sigma)$ are monotonically decreasing functions of σ^2 (Proposition 3, Equation S29), we have that

$$\lim_{\sigma^2\to 0} \max_{\Sigma\succcurlyeq 0, \text{tr}\,\Sigma=1} f_{T,\sigma^2}(\Sigma) = \sup_{\sigma^2\geq 0} \max_{\Sigma\succcurlyeq 0, \text{tr}\,\Sigma=1} f_{T,\sigma^2}(\Sigma) = \max_{\Sigma\succcurlyeq 0, \text{tr}\,\Sigma=1} \sup_{\sigma^2\geq 0} f_{T,\sigma^2}(\Sigma) = \max_{\Sigma\succcurlyeq 0, \text{tr}\,\Sigma=1} \lim_{\sigma^2\to 0} f_{T,\sigma^2}(\Sigma). \tag{S51}$$

If Σ maximizing $\lim_{\sigma^2\to 0} f_{T,\sigma^2}(\Sigma)$ is such that $(e^{AT}, B\Sigma^{1/2})$ is a controllable pair, then $\mathcal{W} > 0$, and we have

$$\begin{aligned}\frac{1}{T}\lim_{\sigma^2\to 0} f_{T,\sigma^2}(\Sigma) &= \frac{1}{2T}\log_2 \frac{\det(\mathcal{O}\mathcal{W})}{\det(\mathcal{O}e^{AT}\mathcal{W}e^{A^\top T})} \\ &= -\frac{1}{2T}\log_2 \det(e^{AT})^2 = -\frac{1}{T\ln 2}\ln\det(e^{AT}) \\ &= -\frac{1}{T\ln 2}\text{tr}(AT) = -\frac{1}{\ln 2}\text{tr}(A),\end{aligned} \tag{S52}$$

where in the second equation we used the fact that $\mathcal{O}$ is invertible, in view of the observability of (A, C). If the maximizing Σ is such that $(e^{AT}, B\Sigma^{1/2})$ is *not* controllable, then, via a suitable similarity transformation Q, we can bring e^{AT} and $B\Sigma^{1/2}$ in Kalman canonical form (*27, Chapter 16*), namely

$$\overline{A} := Q^{-1}e^{AT}Q = \begin{bmatrix} A_{11} & A_{12} \\ 0 & A_{22}\end{bmatrix}, \quad \overline{B} := Q^{-1}B\Sigma^{1/2} = \begin{bmatrix} B_1 \\ 0 \end{bmatrix}, \tag{S53}$$

where (A_{11}, B_1) forms a controllable pair. By partitioning $\mathcal{O}$ conformably to the block partition of $\overline{A}$, namely,

$$\overline{\mathcal{O}} := Q^{-\top}\mathcal{O}Q^{-1} = \begin{bmatrix} \mathcal{O}_{11} & \mathcal{O}_{12} \\ \mathcal{O}_{21} & \mathcal{O}_{22}\end{bmatrix}, \quad \mathcal{O}_{11} > 0, \tag{S54}$$

it follows that,

$$\frac{1}{T}\lim_{\sigma^2\to 0} f_{T,\sigma^2}(\Sigma) = -\frac{1}{T}\log_2\det(A_{11}) < -\frac{1}{T}\log_2\det(e^{AT}), \tag{S55}$$

since the eigenvalues of A_{11} are a subset of eigenvalues of e^{AT}. This in turn implies that the maximum of $\lim_{\sigma^2\to 0} f_{T,\sigma^2}(\Sigma)$ cannot be achieved by any Σ that renders $(e^{AT}, B\Sigma^{1/2})$ not controllable and, therefore, its value is given by Equation S52. □

Theorem 2 (Behavior of $\mathcal{R}_T$ in the high noise regime). *Let $A \in \mathbb{R}^{n\times n}$ be a stable matrix. For all $T > 0$, it holds*

$$\lim_{\sigma^2\to\infty} \sigma^2 \mathcal{R}_T = \frac{1}{2\ln 2T}\|B\mathcal{O}B^\top\|. \tag{S56}$$

Further, as $\sigma^2 \to \infty$, the optimal Σ in Equation S16 is given by $\Sigma^\star = vv^\top/(v^\top v)$, where $v \in \mathbb{R}^m$ is the eigenvector corresponding to the maximum eigenvalue of $B\mathcal{O}B^\top$.

Proof. Since $\sigma^2 f_{T,\sigma^2}(\Sigma)$ and $\max_{\Sigma\succcurlyeq 0,\mathrm{tr}\,\Sigma=1} \sigma^2 f_{T,\sigma^2}(\Sigma)$ are monotonically increasing functions of σ^2 (Proposition 3, Equation S30),

$$\lim_{\sigma^2\to\infty}\max_{\Sigma\succcurlyeq 0,\mathrm{tr}\,\Sigma=1} \sigma^2 f_{T,\sigma^2}(\Sigma) = \sup_{\sigma^2\geq 0}\max_{\Sigma\succcurlyeq 0,\mathrm{tr}\,\Sigma=1} \sigma^2 f_{T,\sigma^2}(\Sigma) = \max_{\Sigma\succcurlyeq 0,\mathrm{tr}\,\Sigma=1}\sup_{\sigma^2\geq 0} \sigma^2 f_{T,\sigma^2}(\Sigma) = \max_{\Sigma\succcurlyeq 0,\mathrm{tr}\,\Sigma=1}\lim_{\sigma^2\to\infty} \sigma^2 f_{T,\sigma^2}(\Sigma). \tag{S57}$$

Using the Taylor expansion of the natural logarithm $\ln(1 + x) = x + \text{h.o.t.}$ as $x \to 0$, it holds

$$\ln\det(I + \mathcal{O}\mathcal{W}/\sigma^2) = \mathrm{tr}(\mathcal{O}\mathcal{W})/\sigma^2 + \text{h.o.t.} \quad \text{as} \quad \sigma^2 \to \infty, \tag{S58}$$

$$\ln\det(I + \mathcal{O}(\mathcal{W} - B^\top\Sigma B)/\sigma^2) = \mathrm{tr}(\mathcal{O}(\mathcal{W} - B^\top\Sigma B))/\sigma^2 + \text{h.o.t.} \quad \text{as} \quad \sigma^2 \to \infty, \tag{S59}$$

so that

$$\begin{aligned}
\lim_{\sigma^2\to\infty}\sigma^2 f_{T,\sigma^2}(\Sigma) &= \frac{1}{2\ln 2}\lim_{\sigma^2\to\infty}\sigma^2\ln\frac{\det(I + \mathcal{O}\mathcal{W}/\sigma^2)}{\det(I + \mathcal{O}(\mathcal{W} - B^\top\Sigma B)/\sigma^2)} \\
&= \frac{1}{2\ln 2}\lim_{\sigma^2\to\infty}\sigma^2\left(\ln\det(I + \mathcal{O}\mathcal{W}/\sigma^2) - \ln\det(I + \mathcal{O}(\mathcal{W} - B^\top\Sigma B)/\sigma^2)\right) \\
&= \frac{1}{2\ln 2}\mathrm{tr}(\mathcal{O}B^\top\Sigma B).
\end{aligned} \tag{S60}$$

Next, we note that the following inequality holds (*54*)

$$\mathrm{tr}(\mathcal{O}B^\top\Sigma B) = \mathrm{tr}(B\mathcal{O}B^\top\Sigma) \leq \|B\mathcal{O}B^\top\|\,\mathrm{tr}(\Sigma). \tag{S61}$$

On the other hand, by picking $\Sigma^\star = vv^\top/(v^\top v)$, with $v \in \mathbb{R}^n$ being the eigenvector corresponding to the maximum eigenvalue of $B\mathcal{O}B^\top$, we have $\mathrm{tr}(B\mathcal{O}B^\top\Sigma^\star) = \|B\mathcal{O}B^\top\|\,\mathrm{tr}(\Sigma^\star) = \|B\mathcal{O}B^\top\|$. Hence,

$$\max_{\Sigma\succcurlyeq 0,\mathrm{tr}\,\Sigma=1}\mathrm{tr}(B\mathcal{O}B^\top\Sigma) = \|B\mathcal{O}B^\top\|, \tag{S62}$$

from which Equation S56 follows using Equation S57. □

5 Information rate of normal networks

We derive here an explicit expression for the information information rate of networks described by a normal adjacency matrix with $B = C = I$. To this end, we first establish an instrumental lemma. In what follows, $\mathrm{diag}(X)$ will denote the diagonal matrix with the diagonal entries of X, if X is a matrix, or the entries of X along the diagonal, if X is a vector.

Lemma 1. *Let $P \in \mathbb{C}^{n\times n}$ be a positive definite Hermitian matrix. It holds* $\mathrm{diag}(P^{-1}) \succcurlyeq (\mathrm{diag}\ P)^{-1}$.

Proof. Let $\{e_i\}_{i=1}^n$ be the canonical basis in $\mathbb{R}^n$, by Cauchy–Schwarz inequality $(e_i^\top Pe_i)(e_i^\top P^{-1}e_i) = \|P^{1/2}e_i\|^2\|P^{-1/2}e_i\|^2 \geq (e_i^\top P^{1/2}P^{-1/2}e_i)^2 = 1$, so that $e_i^\top P^{-1}e_i \geq (e_i^\top Pe_i)^{-1}$ for all $i = 1, \dots, n$. □

Theorem 3 (information rate of normal networks). *If $B = C = I$ and $A \in \mathbb{R}^{n\times n}$ is a normal and stable matrix with eigenvalues $\{\lambda_i\}_{i=1}^n$, then*

$$\mathcal{R}_T = \max_{\substack{\{P_i\}_{i=1}^n, P_i\geq 0 \\ s.t.\ \sum_{i=1}^n P_i = P}}\sum_{i=1}^n \mathcal{R}_T(P_i, \lambda_i), \tag{S63}$$

with

$$\mathcal{R}_T(P,\lambda) := \frac{1}{2T}\log_2 \frac{\frac{P}{\sigma^2} - 2\mathrm{Re}\,\lambda}{\frac{P}{\sigma^2}\, e^{2T\mathrm{Re}\,\lambda} - 2\mathrm{Re}\,\lambda}. \tag{S64}$$

Proof. Consider the function f_{T,σ^2} as previously defined in Equation S25. We will show that the matrix $\Sigma \succcurlyeq 0$, $\mathrm{tr}(\Sigma) = 1$, maximizing $f_{T,\sigma^2}(\Sigma)$ is diagonal w.r.t. the same basis diagonalizing A. Along the lines of the proof of Proposition 2, we first note that f_{T,σ^2} can be equivalently rewritten as

$$f_{T,\sigma^2}(\Sigma) = \frac{1}{2\ln 2}\ln\det\left(I - \sigma^2 K X^{-1}\right) - \frac{T}{\ln 2}\mathrm{tr}(A), \tag{S65}$$

where $X := \mathcal{W} + \sigma^2 e^{-AT}\mathcal{O}^{-1}e^{-A^\top T}$ and $K := e^{-AT}\mathcal{O}^{-1}e^{-A^\top T} - \mathcal{O}^{-1}$. Next, if A is stable and normal, it can be unitarily diagonalized in the form $A = -U^\dagger \Lambda U$, where U is a unitary matrix and Λ is diagonal with diagonal entries $\{\lambda_i\}_{i=1}^n$ having positive real part. Since $C = I$, we can rewrite $\mathcal{O}$ as

$$\mathcal{O} = U^\dagger\left[\frac{1}{2}\mathrm{Re}(\Lambda)^{-1}\left(I - e^{-2\mathrm{Re}(\Lambda)T}\right)\right]U, \tag{S66}$$

where $\mathrm{Re}(\cdot)$ denotes element-wise real part when applied to matrices, so that Equation S65 can be rewritten as

$$f_{T,\sigma^2}(\Sigma) = \frac{1}{2\ln 2}\ln\det\left(I - \sigma^2\overline{K}^{1/2}\overline{X}^{-1}\overline{K}^{1/2}\right) - \frac{T}{\ln 2}\mathrm{tr}(A), \tag{S67}$$

where $\overline{X} := U\mathcal{W}U^\dagger + \frac{\sigma^2}{2}\mathrm{Re}(\Lambda)\left(e^{2\mathrm{Re}(\Lambda)T} - I\right)^{-1}$ and $\overline{K} := \frac{1}{2}\mathrm{Re}(\Lambda)e^{2\mathrm{Re}(\Lambda)T} \succ 0$. Now assume, by contradiction, that the optimal $\Sigma^\star$, $\mathrm{tr}(\Sigma^\star) = 1$, maximizing $f_{T,\sigma^2}(\Sigma)$ yields a matrix $\bar{\Sigma}^\star := U\Sigma^\star U^\dagger$ which is not diagonal. This implies that $Z^\star := \overline{K}^{1/2}\overline{X}^{-1}\overline{K}^{1/2}$ is not diagonal, since

(i) $U\mathcal{W}U^\dagger = \sum_{k=0}^{\infty} e^{-\Lambda kT}\bar{\Sigma}e^{-\Lambda^\dagger kT}$ is not diagonal,

(ii) $\frac{\sigma^2}{2}\mathrm{Re}(\Lambda)\left(e^{2\mathrm{Re}(\Lambda)T} - I\right)^{-1}$ and $\overline{K}$ are diagonal matrices, and

(iii) the inverse of a non-singular, non-diagonal matrix cannot be diagonal.

Let $\bar{\Sigma}_d := \mathrm{diag}(\bar{\Sigma}^\star)$ and consider the corresponding diagonal matrix $Z_d := K^{1/2}\overline{X}_d^{-1}K^{1/2}$, where

$$\overline{X}_d := U\mathcal{W}_d U^\dagger + \frac{\sigma^2}{2}\mathrm{Re}(\Lambda)\left(e^{2\mathrm{Re}(\Lambda)T} - I\right)^{-1}, \tag{S68}$$

and $\mathcal{W}_d := \sum_{k=0}^{\infty} e^{-\Lambda kT}\bar{\Sigma}_d e^{-\Lambda^\dagger kT}$. Since for every positive definite matrix $P \succ 0$ it holds $\mathrm{diag}(P^{-1}) \succcurlyeq (\mathrm{diag}\ P)^{-1}$ (Lemma 1), we have that

$$\mathrm{diag}(Z^\star) = \overline{K}^{1/2}\,\mathrm{diag}(\overline{X}^{-1})\overline{K}^{1/2} \succcurlyeq \overline{K}^{1/2}\bar{X}_d^{-1}\overline{K}^{1/2} = Z_d. \tag{S69}$$

Eventually, we argue that

$$\det(I - Z^\star) < \det(I - \mathrm{diag}(Z^\star)) \overset{eq.\ S69}{\leq} \det(I - Z_d), \tag{S70}$$

where the first inequality follows from the fact that $I - Z^\star \succ 0$ and from Hadamard's inequality $\det P \leq \det\mathrm{diag}(P)$, for a positive definite $P \succ 0$ (with equality attained if and only if P is diagonal, cf. (*55, p. 505*)). The inequality in Equation S70 contradicts the optimality of $\Sigma^\star$ and therefore the matrix $U\Sigma^\star U^\dagger$ must be diagonal. In view of this fact, the desired expression in Equation S63 follows by direct computation. □

From the above result we have the following interesting corollary.

Corollary 1 (Optimal power allocation for normal networks). *If $B = C = I$ and A is normal, then $\mathcal{R}_{\max} := \max_{T\geq 0}\mathcal{R}_T$ is achieved for $T \to 0$ and the optimal power allocation is*

$$P_i = \frac{\mathrm{Re}\,\lambda_i}{\mathrm{tr}\,A}P, \tag{S71}$$

which yields

$$\mathcal{R}_{\max} = \frac{1}{\ln 2}\frac{P\,\mathrm{tr}\,A}{2\sigma^2\,\mathrm{tr}\,A - P}. \tag{S72}$$

Proof. Since $\mathcal{R}_T$ is the sum of n terms of the form in Equation S64 and each of these terms is a monotonically decreasing function of T, it follows that the maximum of $\mathcal{R}_T$ is achieved for $T \to 0$. For $T \to 0$, the expression of $\mathcal{R}_T$ becomes

$$\mathcal{R}_T = \frac{1}{\ln 2} \max_{\substack{\{P_i\}_{i=1}^n, P_i \geq 0 \\ \text{s.t. } \sum_{i=1}^n P_i = P}} \sum_{i=1}^{n} \frac{\frac{P_i}{\sigma^2} \operatorname{Re} \lambda_i}{2 \operatorname{Re} \lambda_i - \frac{P_i}{\sigma^2}}. \tag{S73}$$

Consider the Lagrangian of the above constrained optimization problem

$$\mathcal{L}(P_1, \dots, P_n, \mu) = \sum_{i=1}^{n} \frac{\frac{P_i}{\sigma^2} \operatorname{Re} \lambda_i}{2 \operatorname{Re} \lambda_i - \frac{P_i}{\sigma^2}} + \mu \left(P - \sum_{i=1}^{n} P_i \right), \tag{S74}$$

where $\mu \in \mathbb{R}$ is the Lagrangian multiplier. By equating $\partial \mathcal{L} / \partial P_i$ to zero, we have

$$\frac{\partial \mathcal{L}}{\partial P_i} = \frac{2(\operatorname{Re} \lambda_i)^2}{\left(-2 \operatorname{Re} \lambda_i + \frac{P_i}{\sigma^2}\right)^2} - \mu = 0 \Rightarrow \frac{P_i}{\sigma^2} = \left(2 \pm \sqrt{\frac{2}{\mu}} \right) \operatorname{Re} \lambda_i, \quad i = 1, 2, \dots, n. \tag{S75}$$

In view of the latter equation,

$$\frac{P}{\sigma^2} = \sum_{i=1}^{n} \frac{P_i}{\sigma^2} = \left(2 \pm \sqrt{\frac{2}{\mu}} \right) \sum_{i=1}^{n} \operatorname{Re} \lambda_i = \left(2 \pm \sqrt{\frac{2}{\mu}} \right) \operatorname{tr} A \Rightarrow \frac{P_i}{\sigma^2} = \frac{P}{\operatorname{tr} A} \operatorname{Re} \lambda_i. \tag{S76}$$

Eventually, by substituting the above optimal signal-to-noise ratios P_i/σ^2 into Equation S73, we obtain

$$\mathcal{R}_{\max} = \frac{1}{\ln 2} \frac{P \operatorname{tr} A}{2\sigma^2 \operatorname{tr} A - P}. \tag{S77}$$

which ends the proof. □

It is worth pointing out that, from Proposition 5, Equation S63 provides an upper bound to the achievable rate of any normal network for *any* choice of input and output node subsets. Furthermore, the expression of $\mathcal{R}_{\max}$ in Equation S72 corresponds to the maximum information rate that can be attained by a network described by a normal matrix A for a fixed noise level.

6 Measures of matrix non-normality and network indicators of non-normality

Mathematically speaking, matrix non-normality is not easy to measure and there does not exist a unique scalar parameter that quantifies it. However, several scalar estimates of matrix non-normality have been proposed and studied in the literature, see Chapter 48 of (*20*) for a complete survey. There are two general approaches for quantifying the "degree" of non-normality of a matrix $A \in \mathbb{R}^{n \times n}$. The first and classic approach is to measure non-normality in terms of departure from some algebraic properties characterizing the set of normal matrices. In particular, metrics that fall into this category are zero when evaluated at normal matrices. Examples include:

- the distance from commutativity, $c(A) = \|AA^\top - A^\top A\|_F$, where $\|\cdot\|_F$ is the Frobenius norm;
- the Henrici's departure from normality, $h(A) = (\|A\|_F^2 - \sum_{z \in \lambda(A)} |z|^2)^{1/2}$, where $\lambda(\cdot)$ denotes the set of eigenvalues (or spectrum) of a matrix.

Alternatively, one can consider the linear dynamical system governed by A, and quantify non-normality in terms of the ability of this system to transiently amplify its trajectories. This ability is, in turn, related to the sensitivity of the spectrum of A to perturbations of the entries of A (*20*). Some standard measures of non-normality that carry such a "dynamical" interpretation are:

- the numerical abscissa, $\omega(A) = \max_{z \in \lambda((A+A^\top)/2)} z$;
- the condition number of the eigenvector matrix of A, $\kappa(A) = \|V\| \|V^{-1}\|$, where the columns of V are the eigenvectors of A. (Here, we assume that A is diagonalizable. Note that, if A is normal, then $\kappa(A) = 1$.)

In the network considered in our paper, the above-listed non-normality metrics correlate with some scalar network parameters. These parameters can thus be thought of as knobs regulating the "degree" of non-normality of the network. Specifically, for the

chain network in Equation (9) of the main text, as α (directionality strength) grows, then all of the above-listed measures of non-normality increase as well (Supplementary Figure S1, left plot). For $\alpha \neq 1$, $h(A)$, $\kappa(A)$, and $\omega(A)$ are positively correlated with the chain length ℓ, whereas $c(A)$ is independent of ℓ (Supplementary Figure S1, right plot). A similar correlation behavior is found when considering the more general layered topologies described in Supplementary Note 6 (not shown). Finally, for heterogeneous topologies generated via Equation (11) of the main text, the parameter σ_S (st. dev. of the entries of the skew-symmetric matrix S) and the network dimension n are highly correlated with the non-normality metrics $h(A)$ and $\omega(A)$, whereas they do not exhibit a clear correlation w.r.t. $c(A)$ and $\kappa(A)$ (Supplementary Figure S2).

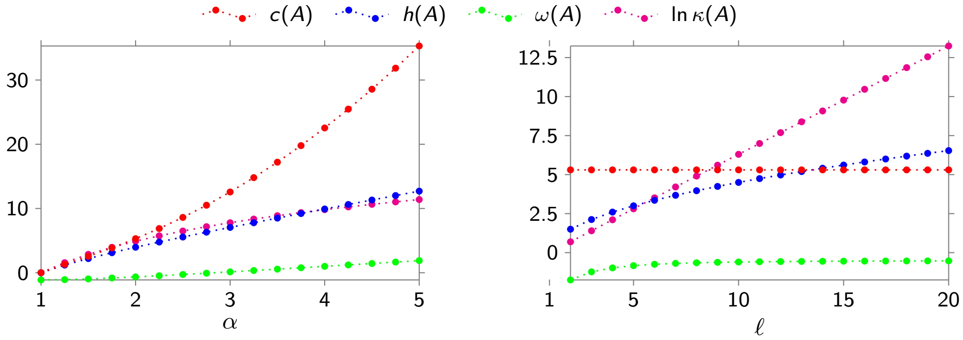

Supplementary Figure S1 | Non-normality metrics and parameters of chain network. Correlation between standard non-normality metrics and parameters α and ℓ of the chain network described in Equation (9) of the main text with $\gamma = -3$ and $\beta = 1$. For better visualization, $\kappa(A)$ is plotted in logarithmic scale. Left plot: $\ell = n = 8$. Right plot: $\ell = n$, $\alpha = 2$.

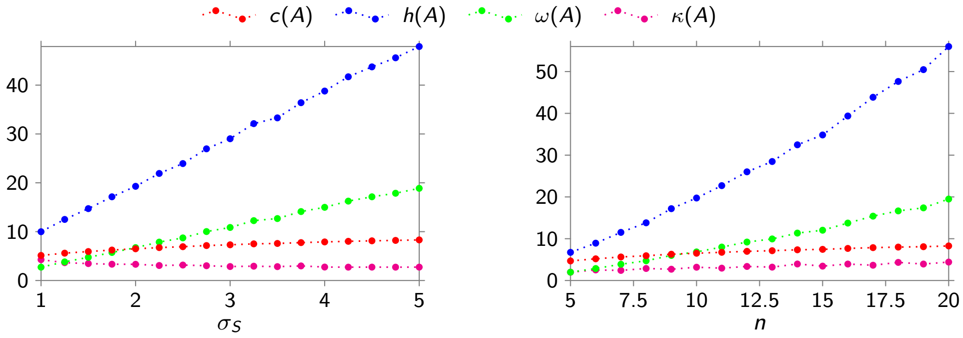

Supplementary Figure S2 | Non-normality metrics and parameters of heterogeneous network. Correlation between standard non-normality metrics and parameter σ_S and dimension n of heterogenous topologies. The curves are the average over 500 realizations of the random model in Equation (11) of the main text with scale parameter $\omega^{-2} = \nu - n - 1$ and $\nu = 24 + n$ degrees of freedom. Left plot: $n = 10$. Right plot: $\sigma_S = 2$.

If matrix A represents the adjacency matrix of a network, then it can be shown that non-normality is related to (i) absence of cycles in the network, (ii) low reciprocity of directed edges, and (iii) presence of hierarchical organization (see (*22*) for further details). With reference to our examples of chain and layered topologies, these networks are clearly acyclic, with reciprocity inversely proportional to α, and number of "hierarchical levels" equal to ℓ.

Finally, we mention that, although for general networks the presence of long directed paths does not necessarily imply (strong) non-normality. For a concrete example, consider a directed ring topology, which is always normal regardless of the directionality strength. However, if the network is stable (a standing assumption in our paper) the connection between strong non-normality (here interpreted in a dynamical sense) and presence of strongly directional network paths can be made more precise. We next provide a simple example of this fact by illustrating what happens to a directed chain in which we add one edge to form a directed ring, while we refer to (*30*) for a more rigorous characterization of the aforementioned connection for general networks with non-negative weights (self-loops excluded). In the example, we consider as non-normality measure the numerical abscissa.

Consider the directed chain described by the following adjacency matrix

$$A = \begin{bmatrix} \gamma & 0 & 0 & & \\ \alpha & \gamma & 0 & \ddots & \\ & \ddots & \ddots & \ddots & 0 \\ & & \ddots & \gamma & 0 \\ & & & \alpha & \gamma \end{bmatrix} \in \mathbb{R}^{n\times n}, \tag{S78}$$

with $\gamma \in \mathbb{R}$ and $\alpha > 0$. It can be shown that the numerical abscissa is

$$\omega(A) = \gamma + \alpha \cos\left(\frac{\pi}{n+1}\right)$$

which, for large n, is approximately equal to

$$\omega(A) \approx \gamma + \alpha.$$

The system described by A exhibits transient amplification if and only if $\omega(A) > 0$ (*20*). If A is stable, then $\gamma < 0$ and the previous condition implies that α should be sufficiently large (strong directionality) for the network to feature a high "degree" of non-normality. If we add an edge so that the directed chain becomes a directed ring, matrix A becomes

$$A' = \begin{bmatrix} \gamma & 0 & 0 & & \alpha \\ \alpha & \gamma & 0 & \ddots & \\ & \ddots & \ddots & \ddots & 0 \\ & & \ddots & \gamma & 0 \\ & & & \alpha & \gamma \end{bmatrix}, \tag{S79}$$

which is indeed a normal matrix with the same trace of A. However, when $\alpha + \gamma > 0$ the largest eigenvalue of A' (which equals its numerical abscissa) is unstable. Thus in the case of strong directionality, the matrix A' becomes unstable, so that the stability assumption is violated.

7 Information rate of a class of non-normal networks

Let $S \in \mathbb{R}^{n\times n}$ be the adjacency matrix of a weighted graph $\mathcal{G} = (\mathcal{V}, \mathcal{E})$, where $\mathcal{V}$ and $\mathcal{E}$ denote the set of nodes and edges of $\mathcal{G}$, respectively, and consider the following matrix

$$A := DSD^{-1}, \tag{S80}$$

where $D := \mathrm{diag}(d_1, d_2, \dots, d_n)$, $d_i > 0$, $i = 1, 2, \dots, n$. Note that the graph with adjacency matrix A has the same connectivity of $\mathcal{G}$ but, in general, different weights. Additionally, A has the same eigenvalues of S, but, typically, a different "degree" of non-normality. The following result characterizes the information rate of the network with adjacency matrix A as in Equation S80.

Theorem 4 (information rate of networks with adjacency matrix $A = DSD^{-1}$). *Let $S \in \mathbb{R}^{n\times n}$ be the stable adjacency matrix of a weighted graph $\mathcal{G} = (\mathcal{V}, \mathcal{E})$, and consider $A := DSD^{-1}$, where $D_1 := \mathrm{diag}(d_1, d_2, \dots, d_n)$, $d_i > 0$, $i = 1, 2, \dots, n$, is a positive diagonal matrix. Let $B = [e_{k_1}, \dots, e_{k_m}]$ and $C = [e_{t_1}, \dots, e_{t_p}]^\top$ be the input matrix and the output matrix respectively and let $d_{\min} := \min\{d_{k_1}, \dots, d_{k_m}\}$ and $d_{\max} = \max\{d_{t_1}, \dots, d_{t_p}\}$. Then,*

$$\mathcal{R}_T(A, \sigma^2) \geq \mathcal{R}_T\left(S, \left(\frac{d_{\min}}{d_{\max}}\sigma\right)^2\right), \tag{S81}$$

where we made explicit the dependence of $\mathcal{R}_T$ on the network adjacency matrix and noise variance.

Proof. Let $r := \arg\min\{d_{k_1}, \dots, d_{k_m}\}$ and $q := \arg\max\{d_{t_1}, \dots, d_{t_p}\}$. Consider first the special case where $C = e_q$ and $B = e_r$. In view of the definition of A, we have $De_q^\top e_q D = d_{\max}^2 e_q^\top e_q$. This in turn implies that

$$\mathcal{O} = \int_0^T e^{A^\top t} C^\top C e^{At} \mathrm{d}t = \int_0^T D^{-1} e^{S^\top t} D e_q^\top e_q D e^{St} D^{-1} \mathrm{d}t = d_{\max}^2 D^{-1} \underbrace{\int_0^T e^{S^\top t} e_q^\top e_q e^{St} \mathrm{d}t}_{=:\widehat{\mathcal{O}}} D^{-1}. \tag{S82}$$

Similarly, we have $D^{-1}e_r e_r^\top D^{-1} = \frac{1}{d_{\min}^2} e_r e_r^\top$, so that we can rewrite $\mathcal{W}$ as

$$\mathcal{W} = \sum_{k=0}^{\infty} e^{AkT} B\Sigma B^\top e^{A^\top kT} = \sum_{k=0}^{\infty} De^{SkT} D^{-1} e_r e_r^\top D^{-1} e^{S^\top kT} D = \frac{1}{d_{\min}^2} D \underbrace{\sum_{k=0}^{\infty} e^{SkT} e_r e_r^\top e^{S^\top kT}}_{=:\widehat{\mathcal{W}}} D. \tag{S83}$$

By substituting Equations S82 and S83 in the expression of $\mathcal{R}_T$, we have

$$\mathcal{R}_T(A, \sigma^2) = \frac{1}{2T} \max_{\Sigma \succcurlyeq 0,\, \mathrm{tr}\,\Sigma = P} \log_2 \frac{\det(\sigma^2 I + \mathcal{O}\mathcal{W})}{\det(\sigma^2 I + \mathcal{O}(\mathcal{W} - B\Sigma B^\top))} = \frac{1}{2T} \max_{\Sigma \succcurlyeq 0,\, \mathrm{tr}\,\Sigma = P} \log_2 \frac{\det(\sigma^2 I + \mathcal{O}\mathcal{W})}{\det(\sigma^2 I + \mathcal{O}e^{AT}\mathcal{W}e^{A^\top T})}$$

$$= \frac{1}{2T} \max_{\Sigma \succcurlyeq 0,\, \mathrm{tr}\,\Sigma = P} \log_2 \frac{\det(\sigma^2 I + \left(\frac{d_{\max}}{d_{\min}}\right)^2 D^{-1}\widehat{\mathcal{O}}D^{-1}D\widehat{\mathcal{W}}D)}{\det(\sigma^2 I + \left(\frac{d_{\max}}{d_{\min}}\right)^2 D^{-1}\widehat{\mathcal{O}}D^{-1}De^{ST}D^{-1}D\widehat{\mathcal{W}}DD^{-1}e^{S^\top T}D)}$$

$$= \frac{1}{2T} \max_{\Sigma \succcurlyeq 0,\, \mathrm{tr}\,\Sigma = P} \log_2 \frac{\det\left(\left(\frac{d_{\min}}{d_{\max}}\sigma\right)^2 I + \widehat{\mathcal{O}}\widehat{\mathcal{W}}\right)}{\det\left(\left(\frac{d_{\min}}{d_{\max}}\sigma\right)^2 I + \widehat{\mathcal{O}}e^{ST}\widehat{\mathcal{W}}e^{S^\top T}\right)} = \mathcal{R}_T\left(S, \left(\frac{d_{\min}}{d_{\max}}\sigma\right)^2\right). \tag{S84}$$

Finally, for the general case where $B = [e_{k_1}, \dots, e_{k_m}]$ is such that $r \in \{k_1, \dots, k_m\}$, and $C = [e_{t_1}, \dots, e_{t_p}]^\top$ is such that $q \in \{t_1, \dots, t_p\}$, the inequality in Equation S85 directly follows from Proposition 5. □

Theorem 4 in particular implies that, as the heterogeneity of the elements of D grows unbounded, we recover the same value of information rate of the *noiseless* case.

Corollary 2 (Limiting behavior of information rate for strongly non-normal networks). *Let B, C and A be as in Theorem 4, and assume that the pair (S, e_q), with $q := \arg\max\{d_{t_1}, \dots, d_{t_p}\}$, is observable. Then,*

$$\lim_{\frac{d_{\min}}{d_{\max}} \to 0} \mathcal{R}_T = -\frac{1}{\ln 2} \mathrm{tr}(A). \tag{S85}$$

Proof. From Theorem 4, decreasing the ratio $d_{\min}/d_{\max}$ is equivalent to decreasing the noise variance σ^2 for a network with adjacency matrix S. Then, Equation S85 directly follows from Theorem 1, noting that $\mathrm{tr}(A) = \mathrm{tr}(S)$. □

From the above analysis, it follows that increasing the non-normality of the network by diminishing the ratio $d_{\min}/d_{\max}$ is always beneficial to the information rate $\mathcal{R}_T$, in that it reduces the detrimental effect of output noise. Indeed, for the class of networks in Equation S80, the ratio $d_{\min}/d_{\max}$, can be thought of regulating the "degree" of non-normality of the network. To clarify this claim, assume that $S_{ij} \neq 0$ and consider two diagonal entries d_i, d_j with $d_i \neq d_j$, in this case the (i, j)-th entry and (j, i)-th entry of A take the form $A_{ij} = \frac{d_i}{d_j} S_{ij}$ and $A_{ji} = \frac{d_j}{d_i} S_{ij}$, respectively. As the ratio d_i/d_j tends either to zero or to infinity, matrix A departs from symmetry because the entries A_{ij} and A_{ji} becomes very heterogenous in magnitude. As a concrete example of this, consider the chain network described in our paper (Equation 9 of the main text). The adjacency matrix of this network (here reported for convenience) reads as

$$A = \begin{bmatrix} \gamma & \beta/\alpha & 0 & \cdots & 0 \\ \alpha\beta & \gamma & \beta/\alpha & \ddots & \vdots \\ 0 & \alpha\beta & \ddots & \ddots & 0 \\ \vdots & \ddots & \ddots & \ddots & \beta/\alpha \\ 0 & \cdots & 0 & \alpha\beta & \gamma \end{bmatrix} \in \mathbb{R}^{n \times n}, \tag{S86}$$

with $\beta, \alpha > 0$ and $\gamma < -2\beta$. As described in our paper and in Supplementary Note 6, the parameter α (directionality strength) regulates the "degree" of non-normality of the network. Notice that the matrix in Equation S86 can be rewritten as in Equation S80 with $D = \mathrm{diag}(1, \alpha, \dots, \alpha^{n-1})$ and

$$S = \begin{bmatrix} \gamma & \beta & 0 & \cdots & 0 \\ \beta & \gamma & \beta & \ddots & \vdots \\ 0 & \beta & \ddots & \ddots & 0 \\ \vdots & \ddots & \ddots & \ddots & \beta \\ 0 & \cdots & 0 & \beta & \gamma \end{bmatrix}. \tag{S87}$$

Assume that $\alpha > 1$. Then, Corollary 2 yields that, as $d_{\min}/d_{\max} = 1/\alpha^{n-1}$ tends to zero, the information rate $\mathcal{R}_T$ tends to $-\frac{1}{\ln 2}\text{tr}(A) = -\frac{\gamma n}{\ln 2}$, which corresponds to the information rate of the network in the noiseless case. In other words, as α as grows unbounded (large directionality strength, and, therefore, strong non-normality), the information rate approaches the one achieved in the noiseless case.

Finally, we stress that a small value of $d_{\min}/d_{\max}$ would typically correspond to a large magnitude of some entries of A, which is not desirable in many scenarios. However, we also observe that, in the above example of the chain network, $d_{\min}/d_{\max}$ depends on the chain length n, and for large n the ratio $d_{\min}/d_{\max}$ can be made small regardless of the value of α (provided that $\alpha > 1$). Inspired by this example, we therefore propose an alternative procedure that leads to a decrease of the ratio $d_{\min}/d_{\max}$, while keeping the entries A bounded in magnitude. We term this procedure "directed stratification" as it partitions the network in different "directed" layers, according to the length of the paths from a given subset of nodes to all the other network's nodes.

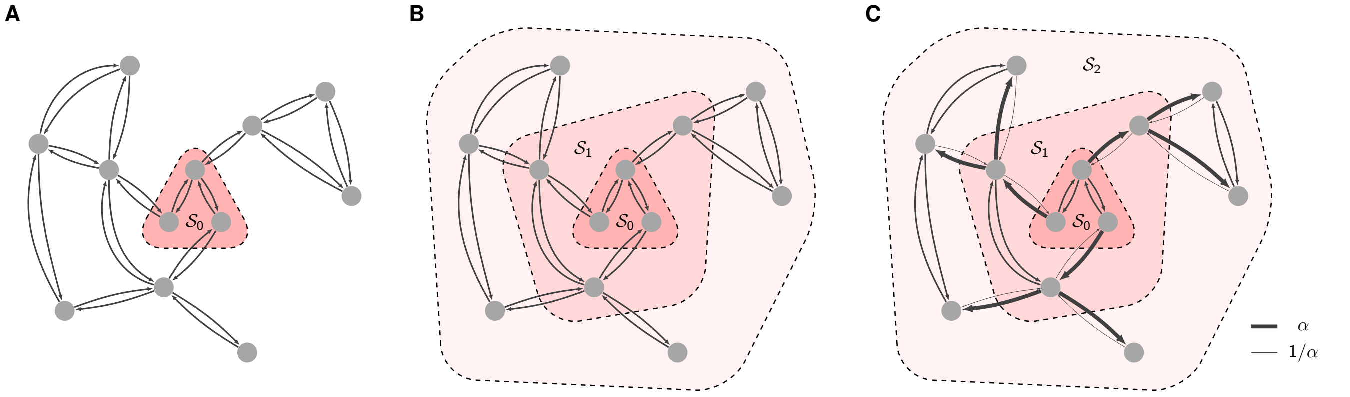

Supplementary Figure S3 | Construction of "layered" non-normal networks. Example of construction of a "layered" non-normal network with $n = 12$ nodes. **(A)** A subset $\mathcal{S}_0$ of nodes in a weights graph $\mathcal{G}$ with homogeneous weights is selected. **(B)** Each node of the network is assigned to a different layers according to its distance from $\mathcal{S}_0$. **(C)** The parameter $\alpha > 0$ is used to generate a directional structure between adjacent layers. The thickness of each edge in the network is proportional to their weight.

To this end, given any two nodes $k, t \in \mathcal{V}$, we denote by $d(k, t)$ the length of a shortest path from the node k to the node t. Given $\mathcal{K} \subseteq \mathcal{V}$ and $t \in \mathcal{V}$, we denote by $d(\mathcal{K}, t)$ the minimum length of a shortest path from the nodes in $\mathcal{K}$ to the node t, namely

$$d(\mathcal{K}, t) = \min \{ d(k, t) \ : \ k \in \mathcal{K} \} . \tag{S88}$$

Further, let $s_{\min}$ and $s_{\max}$ denote the smallest and largest (in magnitude) non-zero entries of S, respectively, and consider the matrix $A := DSD^{-1}$ as in Equation S80. We select a subset of nodes in the network, say $\mathcal{S}_0$, and define the following sets

$$\mathcal{S}_\ell = \{ v \in \mathcal{V} \ : \ d(\mathcal{S}_0, v) = \ell \} , \ \ell = 1, 2, \dots, \ell_{\max}, \tag{S89}$$

where $\ell_{\max}$ is the maximum length of a shortest path from subset $\mathcal{S}_0$ to any other node in $\mathcal{V}$. Next, we set $d_i = \alpha^\ell$, $\alpha > 0$, for each node $i \in \mathcal{S}_\ell$. Then, for every edge $(i, j) \in \mathcal{E}$, we distinguish three cases:

(i) if $i, j \in \mathcal{S}_\ell$, then $A_{ij} = \frac{d_j}{d_i} S_{ij} = \frac{\alpha^\ell}{\alpha^\ell} S_{ij} = S_{ij}$;

(ii) if $i \in \mathcal{S}_{\ell-1}$ and $j \in \mathcal{S}_\ell$, then $A_{ij} = \frac{\alpha^{\ell-1}}{\alpha^\ell} S_{ij} = \frac{1}{\alpha} S_{ij}$;

(iii) if $i \in \mathcal{S}_\ell$ and $j \in \mathcal{S}_{\ell-1}$, then $A_{ij} = \frac{\alpha^\ell}{\alpha^{\ell-1}} S_{ij} = \alpha S_{ij}$.

From the above equations, it follows that the magnitude of the entries of A in Equation S80 is bounded in the interval $[\min\{s_{\min}/\alpha, \alpha s_{\min}\}, \max\{s_{\max}/\alpha, \alpha s_{\max}\}]$. In graphical terms, the above-described procedure corresponds to a "directed stratification" of $\mathcal{G}$, as illustrated in Supplementary Figure S3.

Finally, if we consider a matrix A generated as above, an input matrix $B = [e_{k_1}, \dots, e_{k_m}]$, such that there exists $r \in \{k_1, \dots, k_m\}$ with $r \in \mathcal{S}_0$, and an output matrix $C = [e_{t_1}, \dots, e_{t_p}]^\top$, such that there exists $q \in \{k_1, \dots, k_m\}$ with $q \in \mathcal{S}_{\ell_{\max}}$, in view of Theorem 4,

$$\mathcal{R}_T(A, \sigma^2) \geq \mathcal{R}_T \left(S, \left(\alpha^{\ell_{\max}-1}\sigma\right)^2 \right) . \tag{S90}$$

The latter equation shows that it is possible to reduce the effect of noise in the rate either by increasing α or, for a fixed $\alpha > 1$, by increasing $\ell_{\max}$. In particular, via a proper tuning of these two parameters, it is possible to increase the rate while keeping the

entries of A bounded in magnitude in a desired interval. Remarkably, this offers a viable way to enhance the communication performance of real-world networks.

8 Optimal communication architectures

Optimal communication networks can be computed by solving the following optimization problem (here, we consider the information rate $\mathcal{R}_T$ as the optimization cost, but a similar analysis can be carried out when choosing $\mathcal{C}_T$):

$$\max_{A\in\mathcal{S}} \mathcal{R}_T(A), \tag{S91}$$

where $\mathcal{S}$ denotes the set of real stable matrices, and we made explicit the dependence of $\mathcal{R}_T$ on A. This problem is non-convex and computationally expensive. In fact, it requires solving two (typically non-convex) optimization problems: one for computing the information rate, and one for finding the optimal A. However this problem can be made more tractable by introducing some mild assumptions and using the properties of the information rate, as we describe next.

In what follows, we restrict the attention to the subset $\mathcal{S}_r \subset \mathcal{S}$ consisting of stable matrices with real eigenvalues and we consider $B = C = I$. For $C = I$, it is not difficult to see that $\mathcal{R}_T$ is invariant under unitary similarity transformation of A. In view of this fact, we can consider the Schur form of the matrices in $\mathcal{S}_r$ and reduce the original problem to an optimization problem over the set of lower triangular matrices with negative diagonal entries. We denote the latter set with $\mathcal{S}_{\Delta,r}$. The new "simplified" optimization problem reads as

$$\max_{A\in\mathcal{S}_{\Delta,r}} \mathcal{R}_T(A) - \varepsilon\|A\|_F, \tag{S92}$$

where we added a regularization term $\varepsilon\|A\|_F$, $\varepsilon > 0$, to the objective function $\mathcal{R}_T(A)$ to bound the magnitude of the entries of A. Recall that the computation of the information rate $\mathcal{R}_T$ requires solving a constrained optimization problem over input covariances Σ. Consequently, in order to numerically solve the problem in Equation S92, we exploited a coordinate gradient ascent strategy (*56, Ch. 9*) over unit-trace covariance matrices Σ, and connectivity matrices $A \in \mathcal{S}_{\Delta,r}$. Numerical solutions of the optimization problem in Equation S92 are shown in Supplementary Figure S4, for a network of 10 nodes, $\sigma^2 = 1$, and different values of $T > 0$.

From these plots, we observe that:

1. for small values of T, the optimal structure $A^\star$ is diagonal and, therefore, normal. In this case, the corresponding optimal input covariance $\Sigma^\star$ is also diagonal.
2. as T increases, the strictly lower triangular entries of $A^\star$ becomes different from zero, yielding a non-normal optimal network structure. In particular, for T large enough, the entries of the subdiagonal of $A^\star$ are greater than the other lower triangular entries, resulting in an optimal structure similar to a purely feedforward chain. In this case, the non-zero entries of the corresponding optimal input covariances are localized around the upper diagonal entries (source nodes).

To investigate the effect of noise on the optimal matrix, we further optimized the cost function in Equation S92 over the transmission window $T \geq 0$. Namely, we considered the following regularized optimization problem:

$$\max_{A\in\mathcal{S}_{\Delta,r}, T\geq 0} \mathcal{R}_T(A) - \varepsilon\|A\|_F, \tag{S93}$$

As before, we exploited a coordinate gradient ascent method to solve the latter problem. In this case, gradient ascent need to be jointly performed w.r.t. unit-trace covariances Σ, lower triangular connectivity matrices $A \in \mathcal{S}_{\Delta,r}$, and non-negative transmission windows $T \geq 0$. Supplementary Figure S5 shows the numerical results obtained via this optimization strategy, for different values of σ^2. We notice that, for small values of σ^2, the optimal transmission window equals zero, and the optimal connectivity matrix and covariance are both diagonal. For larger values of σ^2 the optimal transmission window becomes strictly positive, and grows as σ^2 increases. Further, the resulting $A^\star$ is non-normal and resembles a feedforward chain, while $\Sigma^\star$ has non-zero diagonal entries concentrated around the upper diagonal entries.

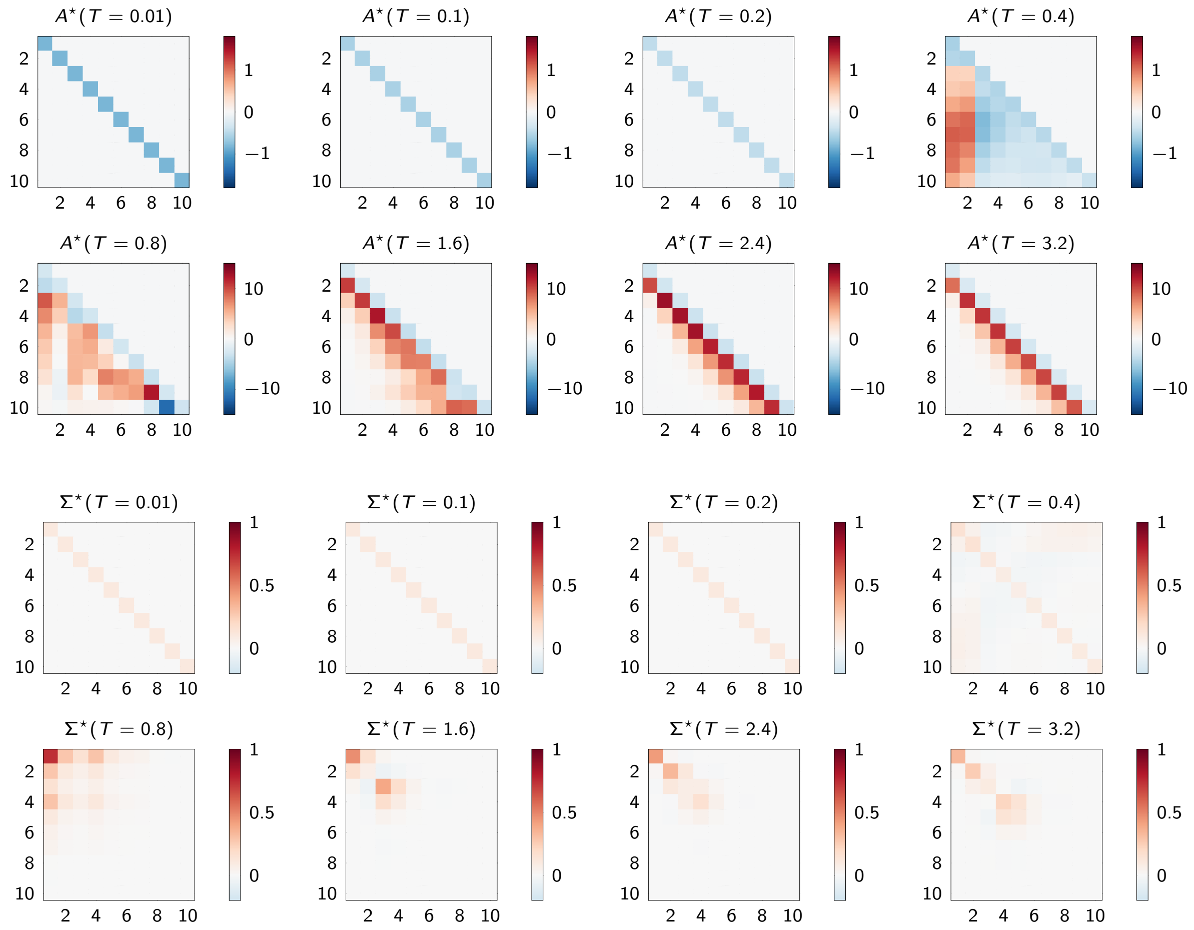

Supplementary Figure S4 | Optimal networks and input covariances as a function of the transmission window T. Optimal network architecture $A^{\star}$ and input covariance $\Sigma^{\star}$ obtained by solving the optimization problem in Equation S93 for $n = 10$, $\sigma^2 = 1$, $\varepsilon = 2.5 \times 10^{-3}$. The solution of the optimization problem has been computed via unconstrained coordinate gradient ascent over unit-trace positive definite Σ and lower triangular $A \in \mathcal{S}_{\Delta,r}$. The simulations have been carried out in Python using Autograd *(45)*.

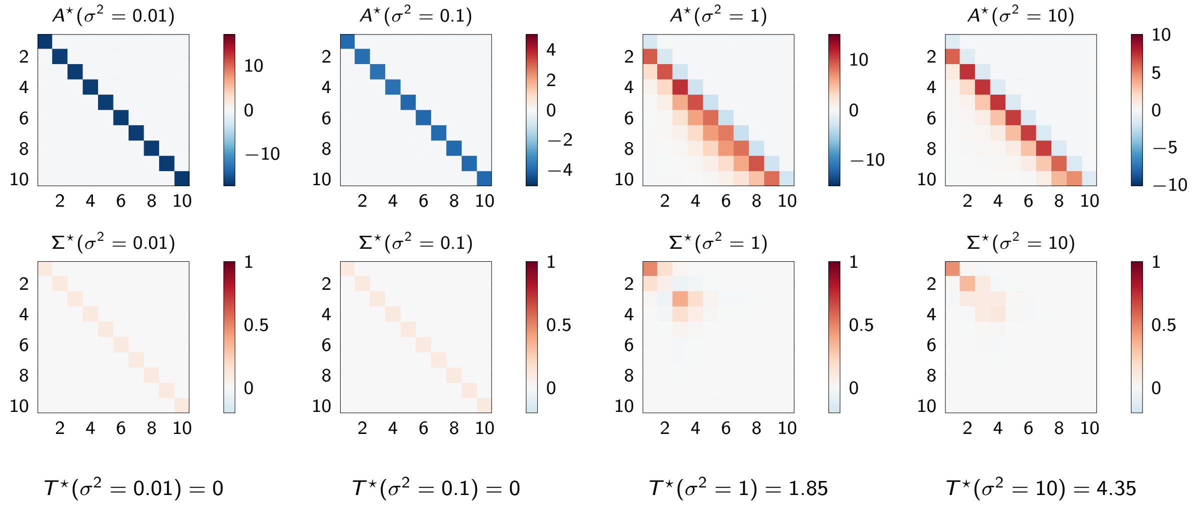

Supplementary Figure S5 | Optimal networks and input covariances as a function of the noise covariance σ^2. Optimal network architecture $A^{\star}$, input covariance $\Sigma^{\star}$, and transmission window $T^{\star}$ obtained by solving the problem in Equation S93 for $n = 10$ nodes, $\varepsilon = 2.5 \times 10^{-3}$, and different values of σ^2. The solution of the problem in Equation S93 has been computed via unconstrained coordinate gradient ascent over unit-trace positive definite Σ's, lower triangular $A \in \mathcal{S}_{\Delta,r}$, and non-negative T.

REFERENCES AND NOTES

1. A. Guille, H. Hacid, C. Favre, D. A. Zighed, Information diffusion in online social networks: A survey. *ACM SIGMOD Rec.* **42**, 17–28 (2013).

2. S. Molaei, S. Babaei, M. Salehi, M. Jalili, Information spread and topic diffusion in heterogeneous information networks. *Sci. Rep.* **8**, 9549 (2018).

3. C. Decker, R. Wattenhofer, Information propagation in the Bitcoin network, in *Proceedings of the 2013 IEEE Thirteenth International Conference on Peer-to-Peer Computing (P2P)* (2013), pp. 1–10.

4. R. Cheong, A. Rhee, C. J. Wang, I. Nemenman, A. Levchenko, Information transduction capacity of noisy biochemical signaling networks. *Science* **334**, 354–358 (2011).

5. J. Selimkhanov, B. Taylor, J. Yao, A. Pilko, J. Albeck, A. Hoffmann, L. Tsimring, R. Wollman, Accurate information transmission through dynamic biochemical signaling networks. *Science* **346**, 1370–1373 (2014).

6. S. Galli, A. Scaglione, Z. Wang, For the grid and through the grid: The role of power line communications in the smart grid. *Proc. IEEE* **99**, 998–1027 (2011).

7. S. B. Laughlin, T. J. Sejnowski, Communication in neuronal networks. *Science* **301**, 1870–1874 (2003).

8. A. Avena-Koenigsberger, B. Misic, O. Sporns, Communication dynamics in complex brain networks. *Nat. Rev. Neurosci.* **19**, 17–33 (2018).

9. T. Toyoizumi, J.-P. Pfister, K. Aihara, W. Gerstner, Generalized Bienenstock–Cooper–Munro rule for spiking neurons that maximizes information transmission. *Proc. Natl. Acad. Sci. U.S.A.* **102**, 5239–5244 (2005).

10. T. Sharpee, W. Bialek, Neural decision boundaries for maximal information transmission. *PLOS ONE* **2**, e646 (2007).

11. E. R. Kandel, J. H. Schwartz, T. M. Jessell, S. A. Siegelbaum, A. J. Hudspeth, *Principles of Neural Science* (McGraw-Hill New York, 2000).

12. D. L. K. Yamins, J. J. DiCarlo, Using goal-driven deep learning models to understand sensory cortex. *Nat. Neurosci.* **19**, 356–365 (2016).

13. R. Shwartz-Ziv, N. Tishby, Opening the black box of deep neural networks via information. arXiv:1703.00810 (2017).

14. I. Goodfellow, Y. Bengio, A. Courville, *Deep Learning* (MIT Press, 2016).

15. N. Tishby, F. C. Pereira, W. Bialek, The information bottleneck method. arXiv:physics/0004057 (2000).

16. N. Rubido, C. Grebogi, M. S. Baptista, Understanding information transmission in complex networks. arXiv:1705.05287 (2017).

17. C. Kirst, M. Timme, D. Battaglia, Dynamic information routing in complex networks. *Nat. Commun.* **7**, 11061 (2016).

18. U. Harush, B. Barzel, Dynamic patterns of information flow in complex networks. *Nat. Commun.* **8**, 2181 (2017).

19. S. Ganguli, D. Huh, H. Sompolinsky, Memory traces in dynamical systems. *Proc. Natl. Acad. Sci. U.S.A.* **105**, 18970–18975 (2008).

20. L. N. Trefethen, M. Embree, *Spectra and Pseudospectra: The Behavior of Nonnormal Matrices and Operators* (Princeton Univ. Press, 2005).

21. M. Asllani, T. Carletti, Topological resilience in non-normal networked systems. *Phys. Rev. E* **97**, 042302 (2018).

22. M. Asllani, R. Lambiotte, T. Carletti, Structure and dynamical behavior of non-normal networks. *Sci. Adv.* **4**, eaau9403 (2018).

23. P. Dayan, L. F. Abbott, *Theoretical Neuroscience* (Cambridge, MIT Press, MA, 2001), vol. 806 of Computational Neuroscience Series.

24. H. K. Inagaki, L. Fontolan, S. Romani, K. Svoboda, Discrete attractor dynamics underlies persistent activity in the frontal cortex. *Nature* **566**, 212–217 (2019).

25. B. Sklar, *Digital Communications: Fundamentals and Applications* (Prentice Hall Upper Saddle River, ed. 2, 2001).

26. T. M. Cover, J. A. Thomas, *Elements of Information Theory* (John Wiley & Sons, 2012).

27. J. P. Hespanha, *Linear Systems Theory* (Princeton Univ. Press, 2009).

28. E. Castet, G. S. Masson, Motion perception during saccadic eye movements. *Nat. Neurosci.* **3**, 177–183 (2000).

29. R. Ben-Yishai, R. L. Bar-Or, H. Sompolinsky, Theory of orientation tuning in visual cortex. *Proc. Natl. Acad. Sci. U.S.A.* **92**, 3844–3848 (1995).

30. G. Baggio, S. Zampieri, in On the relation between non-normality and diameter in dynamical networks, *European Control Conference (ECC)* (IEEE, 2018), pp. 1839–1844.

31. M. S. Goldman, Memory without feedback in a neural network. *Neuron* **61**, 621–634 (2009).

32. B. K. Murphy, K. D. Miller, Balanced amplification: A new mechanism of selective amplification of neural activity patterns. *Neuron* **61**, 635–648 (2009).

33. G. Hennequin, T. P. Vogels, W. Gerstner, Non-normal amplification in random balanced neuronal networks. *Phys. Rev. E* **86**, 011909 (2012).

34. G. Hennequin, L. Aitchison, M. Lengyel, Fast sampling-based inference in balanced neuronal networks, *Advances in Neural Information Processing Systems* (2014), pp. 2240–2248.

35. R. Muolo, M. Asllani, D. Fanelli, P. K. Maini, T. Carletti, Patterns of non-normality in networked systems. *J. Theor. Biol.* **480**, 81–91 (2019).

36. D. Fanelli, F. Ginelli, R. Livi, N. Zagli, C. Zankoc, Noise-driven neuromorphic tuned amplifier. *Phys. Rev. E* **96**, 062313 (2017).

37. S. Nicoletti, N. Zagli, D. Fanelli, R. Livi, T. Carletti, G. Innocenti, Non-normal amplification of stochastic quasicycles. *Phys. Rev. E* **98**, 032214 (2018).

38. S. Nicoletti, D. Fanelli, N. Zagli, M. Asllani, G. Battistelli, T. Carletti, L. Chisci, G. Innocenti, R. Livi, Resilience for stochastic systems interacting via a quasi-degenerate network. *Chaos* **29**, 083123 (2019).

39. L. R. Varshney, B. L. Chen, E. Paniagua, D. H. Hall, D. B. Chklovskii, Structural properties of the Caenorhabditis elegans neuronal network. *PLOS Comput. Biol.* **7**, e1001066 (2011).

40. WormAtlas, www.wormatlas.org [accessed 5 January 2020].

41. F. Pasqualetti, C. Favaretto, S. Zhao, S. Zampieri, Fragility and controllability tradeoff in complex networks, *Annual American Control Conference (ACC)* (2018), pp. 216–221.

42. T. Toyoizumi, Nearly extensive sequential memory lifetime achieved by coupled nonlinear neurons. *Neural Comput.* **24**, 2678–2699 (2012).

43. S. Ganguli, H. Sompolinsky, Short-term memory in neuronal networks through dynamical compressed sensing, *Advances in Neural Information Processing Systems* (2010), pp. 667–675.

44. J. Townsend, N. Koep, S. Weichwald, Pymanopt: A python toolbox for optimization on manifolds using automatic differentiation. *J. Mach. Learn. Res.* **17**, 1–5 (2016).

45. D. Maclaurin, D. Duvenaud, R. P. Adams, in Autograd: effortless gradients in numpy, *ICML 2015 AutoML Workshop* (2015).

46. L. F. Abbott, K. Rajan, H. Sompolinsky, *The Dynamic Brain: An Exploration of Neuronal Variability and its Functional Significance*, M. Ding, D. Glanzman, Eds. (Oxford Univ. Press, 2011), chap. 4, pp. 65–82.

47. T. C. Ferrée, S. R. Lockery, Computational rules for chemotaxis in the nematode *C. elegans*. *J. Comput. Neurosci.* **6**, 263–277 (1999).

48. M. Nicoletti, A. Loppini, L. Chiodo, V. Folli, G. Ruocco, S. Filippi, Biophysical modeling of *C. elegans* neurons: Single ion currents and whole-cell dynamics of AWC^{on} and RMD. *PLOS ONE* **14**, e0218738 (2019).

49. F. Pasqualetti, S. Zampieri, F. Bullo, Controllability metrics, limitations and algorithms for complex networks. *IEEE Trans. Control Netw. Syst.* **1**, 40–52 (2014).

50. N. Bof, G. Baggio, S. Zampieri, On the role of network centrality in the controllability of complex networks. *IEEE Trans. Control Netw. Syst.***4** , 643–653 (2017).

51. Y.-Y. Liu, A.-L. Barabási, Control principles of complex systems. *Rev. Mod. Phys.* **88**, 035006 (2016).

52. S. N. Diggavi, T. M. Cover, The worst additive noise under a covariance constraint. *IEEE Trans. Inf. Theory* **47**, 3072–3081 (2001).

53. R. Bhatia, *Matrix Analysis* (Springer, 2013), vol. 169 of Graduate Texts in Mathematics.

54. Y. Fang, K. A. Loparo, X. Feng, Inequalities for the trace of matrix product. *IEEE Trans. Automat. Contr.* **39**, 2489–2490 (1994).

55. R. A. Horn, C. R. Johnson, *Matrix Analysis* (Cambridge Univ. Press, 2012).

56. J. Nocedal, S. Wright, *Numerical Optimization* (Springer, ed. 2, 2006), Springer Series in Operations Research and Financial Engineering.
